# Production method of the Königsaue birch tar documents cumulative culture in Neanderthals

**DOI:** 10.1007/s12520-023-01789-2

**Published:** 2023-05-22

**Authors:** Patrick Schmidt, Tabea J. Koch, Matthias A. Blessing, F. Alexandros Karakostis, Katerina Harvati, Veit Dresely, Armelle Charrié-Duhaut

**Affiliations:** 1 Early Prehistory and Quaternary Ecology, Department of Geosciences, Eberhard Karls University of Tübingen, Tübingen, Germany; 2 Applied Mineralogy, Department of Geosciences, Eberhard Karls University of Tübingen, Tübingen, Germany; 3 DFG Centre for Advanced Studies “Words, Bones, Genes, Tools”, Eberhard Karls University of Tübingen, Tübingen, Germany; 4 Paleoanthropology, Institute for Archaeological Sciences and Senckenberg Centre for Human Evolution and Palaeoenvironment, Department of Geosciences, Eberhard Karls University of Tübingen, Tübingen, Germany; 5 State Office for Heritage Management and Archaeology Saxony-Anhalt – State Museum of Prehistory, Halle, Germany; 6 Laboratoire de Spectrométrie de Masse Des Interactions Et Des Systèmes (LSMIS), Strasbourg University, CNRS, UMR 7140, Strasbourg, CMC France

**Keywords:** Modern behaviours, Cognitive complexity, Early pyrotechnology, Adhesives, Transformative technologies

## Abstract

Birch tar is the oldest synthetic substance made by early humans. The earliest such artefacts are associated with Neanderthals. According to traditional interpretations, their study allows understanding Neanderthal tool behaviours, skills and cultural evolution. However, recent work has found that birch tar can also be produced with simple processes, or even result from fortuitous accidents. Even though these findings suggest that birch tar per se is not a proxy for cognition, they do not shed light on the process by which Neanderthals produced it, and, therefore, cannot evaluate the implications of that behaviour. Here, we address the question of how tar was made by Neanderthals. Through a comparative chemical analysis of the two exceptional birch tar pieces from Königsaue (Germany) and a large reference birch tar collection made with Stone Age techniques, we found that Neanderthals did not use the simplest method to make tar. Rather, they distilled tar in an intentionally created underground environment that restricted oxygen flow and remained invisible during the process. This degree of complexity is unlikely to have been invented spontaneously. Our results suggest that Neanderthals invented or developed this process based on previous simpler methods and constitute one of the clearest indicators of cumulative cultural evolution in the European Middle Palaeolithic.

**Supplementary information:**

The online version contains supplementary material available at 10.1007/s12520-023-01789-2.

## Introduction

One of the earliest known instances of early humans using fire to produce substances otherwise not existing in nature was when Neanderthals made adhesives from birch bark. Although the oldest physical evidence is extremely sparse, this tradition may reach back as far as ~ 200 ka (thousand years) ago (Mazza et al. 2006). The later part of the European Middle Palaeolithic (~ 300–50 ka, also see Table S1) documents tar making at several other Neanderthal sites (Grünberg 2002; Niekus et al. 2019). This finding has implications for our understanding of Neanderthal cognitive evolution because birch trees do not show any visible exudate that could have been recognized as a potential adhesive. To make glue from birch, the bark must be processed using a transformative process (Kurzweil and Todtenhaupt 1992; Weiner 1988). To date, it remains unknown which technology was used for this. Most researchers supposed laborious methods involving underground processes that restrict oxygen flow (Koller et al. 2001; Kozowyk et al. 2017; Schenck and Groom 2016). This belief derived from experiments showing that heat treatment of birch bark in low-oxygen conditions allowed to make birch tar, in fact, that low-oxygen conditions were necessary to make tar (Groom et al. 2013; Palmer 2007; Weiner 1988). Following this interpretation, archaeologists understood birch tar as one of the best proxies pinpointing evolutionary concepts like cognitive complexity (Wragg Sykes 2015) or Neanderthal’s ability to invent complex technology (Niekus et al. 2019; Roebroeks and Soressi 2016). Indeed, most underground techniques producing low-oxygen conditions are resource consuming and difficult (Schmidt 2021). Much of the energy of the used wood fuel is lost in such processes (Brodard et al. 2015) and a certain degree of temperature control is necessary (Koch and Schmidt 2021), potentially lowering the expected success rate. Thus, birch tar may document advanced technology, forward planning and cultural capacity in Neanderthals (Roebroeks and Soressi 2016).

This interpretation has recently been challenged by the finding that there is an alternative pathway for the production of birch tar (Schmidt et al. 2019). It was shown that tar condenses on the surface of stones from burning birch bark. From there, it can be collected by scraping. The process takes place aboveground and can be triggered accidentally when a fire is lit with burning birch bark (a natural tinder). Although no claim was made that Neanderthals actually produced birch tar with this condensation method (Schmidt et al. 2019); its discovery put into question our view that birch tar documents any cognitive processes per se. Tar making with the condensation method does not require imagination because processes take place aboveground and are visible, it has a high success rate (Blessing and Schmidt 2021), i.e. it is not difficult, and tar made this way might even be the result of an accidentally triggered process. Thus, to continue to use birch tar for understanding the behaviour of Neanderthals, it must be demonstrated how the tar was made.

**Fig. 1:**
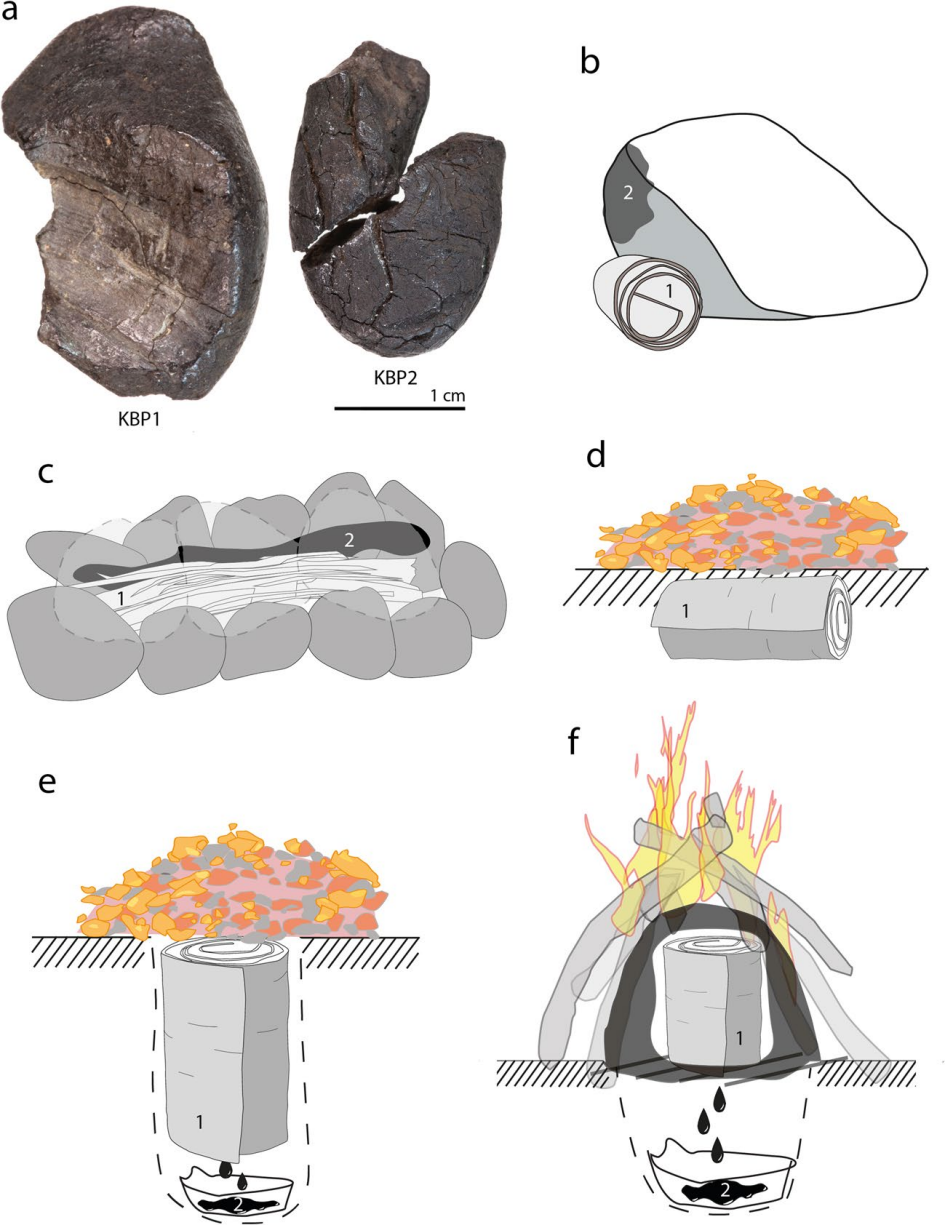
Königsaue birch tar and experimental production techniques. **a** KBP1, Königsaue 1 (left); KBP2, Königsaue 2 (right). **b** Drawing of the condensation method; **c** cobble-groove condensation method; **d** the bark roll buried technique; **e** the pit roll technique; **f** raised structure. 1, birch bark; 2, birch tar. Explanations in the main text but also see supplementary information

In this paper, we investigate the technique Neanderthals used to make tar, to help settle the question of how archaeologists may interpret early tar making in the European Middle Palaeolithic. For this, we analyse the two birch tar artefacts found at the German site Königsaue (Fig. 1a). The pieces weighed 1.35 g and 0.83 g before our analyses; the smaller one is broken in two pieces and both are curated at the Landesmuseum für Vorgeschichte in Halle (Germany). The Königsaue site, excavated in the 1960s (Mania and Toepfer 1973), is located in an open pit soft coal mine that brought to light sediments of a paleo-lake. Neanderthals camped at the shore of this lake producing a site (Picin 2016) that yielded three archaeological horizons. The technocomplexes identified at the site (Mousterian and Micoquian) are highly suggestive of a Neanderthal occupation but a more exact chronological assignment of the site is not straightforward. While Königsaue can unambiguously be assigned to the Middle Palaeolithic, the exact date of the layers from which the two birch tar artefacts were recovered is debated (Grünberg et al. 1999; Mania and Toepfer 1973; Picin 2016). What is certain is that both tars artefacts dated between 45 and 80 ka, not allowing further resolution in terms of their absolute chronology. The larger Königsaue 1 piece was found in a layer below the smaller Königsaue 2 artefact and is thus, at least, relatively older. It should be noted that the layer where the Königsaue 1 piece was found (layer A at Königsaue) is associated with different cultural material (Micoquian) than the layer of the Königsaue 2 piece (which is from level B, assigned to the Mousterian). Thus, there may be a substantial time difference between both pieces. If these two pieces were made with an aboveground method like the condensation method, it would be difficult to argue that Neanderthal birch tar reflects complex technology (Wragg Sykes 2015) because they might be the results of an accidental discovery that was subsequently repeated. If, however, the Königsaue pieces were made with a method including invisible underground processes and intentionally created low-oxygen environments, such a finding would imply that Neanderthals *invented* or *developed* a technical process for transforming their material world. This, in turn, would provide valuable insight into their cognitive and cultural capabilities.

To investigate the tar-making method used by the Königsaue Neanderthals, we produce a reference collection of birch tar made with the most common Stone Age techniques described in the experimental literature as having been used successfully (Groom et al. 2013; Koch and Schmidt 2022; Kozowyk et al. 2017; Palmer 2007; Pomstra and Meijer 2010; Schenck and Groom 2016; Schmidt et al. 2019). We compare their chemical fingerprint with the two tar artefacts from Königsaue to understand which of the experimental tars is most similar to the artefacts.

## Methods and materials

Both artefacts were sampled with a scalpel to obtain powders for the analyses. The larger Königsaue 1 artefact was sampled on its lower side (as oriented in Fig. 1a) that is not shown in the exhibition showcase. The smaller Königsaue 2 artefact was sampled on a surface created by a recent fracture of the piece. Our sampling in the Königsaue 2 artefact left behind a hole that remains invisible if both fragments are shown refitted as they looked before the recent fracture occurred.

### Experimental birch tar making

We conducted an experimental programme to produce a reference collection for our comparative chemical study. We made tar with five different techniques, using only materials available to Neanderthals. The first technique used is the condensation method (Schmidt et al. 2019) (producing 20 samples in separate runs), where the bark is burned beside cobbles to let tar condense on the stone surface (Fig. 1b, Fig. S1). We also made tar using the cobble-groove (Koch and Schmidt 2022) (11 samples) where the bark is burned in an elongated structure lined with flat river cobbles (Fig. 1c, Fig. S2). After the bark burned, tar can be scraped from the inside of the cobbles. These two techniques can be expected to allow relatively good oxygen flow, the fully open-air condensation method likely allowing most oxygen to be available during tar formation. We also employed three underground techniques where bark is heated by a separate fire (as opposed to the bark itself burning). We buried lying bark rolls under thin layers of sediment, building mounds that then were covered with embers (5 samples, Fig. 1d, Fig. S4) (Schenck and Groom 2016). Tar forms in the windings of the rolls. In the second underground approach, we made tar with the pit-roll technique (Kozowyk et al. 2017) (7 samples) where bark rolls are put upright in small pits (Fig. 1e, Fig. S3). Embers are placed on the upper side of the roll and tar drips into a receptacle at the bottom of the pit. The third underground technique approximated a double-pot distillation apparatus (Kurzweil and Todtenhaupt 1992) in aceramic conditions (i.e. without the use of ceramics). Similar techniques have been called ‘raised structures’ (Kozowyk et al. 2017). For this, we heated bark rolls in an upper chamber made from sediment with a surrounding fire, allowing tar to drip into a lower chamber that is separated by a grate (18 samples, Fig. 1f, Fig. S5). These three underground techniques can be expected to restrict oxygen flow, the sealed raised structure likely producing the most reducing conditions (a more detailed description of the five experimental techniques can be found in the Supplementary Information). This experimental program allowed us to produce 61 birch tar samples.

### Infrared spectroscopy

Infrared spectra were recorded from KBr pellets by direct transmission (in a vacuum chamber at > 4 hPa), using a Bruker VERTEX 80v spectrometer, spectral acquisition between 4000 and 400 cm^−1^ and a resolution of 2 cm^−1^. Each ~ 0.3 g pellet contained 0.7 mg of sample. We performed Principal component analysis (PCA), using a covariance matrix, on the first derivative data of the 1800 to 400 cm^−1^ spectral range (yielding 1454 variables). All spectra were first normalised to the highest and lowest points of their CH_2_ and CH_3_ stretching bands in the 3100–2700 cm^−1^ range (after baseline correction of the whole spectra), to reduce remaining differences due to variation in the 0.7 mg samples. Then, the first derivative was calculated over 5 spectral points in the spectral range between 1800 and 400 cm^−1^ to obtain data representative of positive and negative slopes on the spectra that are only minimally influenced by band height.

### Gas chromatography–mass spectrometry (GC–MS)

Molecular analysis was carried out on tar samples that were leftover from our Infrared spectroscopic analysis (sample amounts: KBP1 < 1 mg, KBP2 3 mg). Ten milligrams of reference tars, made with the aboveground condensation method (CM) and the underground raised structure (RS), was used. All samples were processed by ultrasonic-assisted extraction (three times) by means of a mixture dichloromethane/methanol 60:40 v/v (500 µl per extraction step). After concentration under gentle nitrogen flow, the so-obtained organic extract was filtered through diatomaceous earth to remove insoluble residues (elution with dichloromethane/methanol 60:40 v/v). The elution fraction was again concentrated under nitrogen flow to dryness and then engaged in a trimethylsilylation reaction. After the addition of 40 µl pyridine and 200 µl BSTFA, the reaction medium was heated for 2 h at 70 °C and then evaporated to dryness. These silylated organic extracts were dissolved in dichloromethane (20 µl for KBP1 and KBP2, 400 µl for CM and RS) before being injected (2 µl injected). GC–MS analyses were performed with an Agilent 8890 chromatographer coupled with an Agilent 5977B MSD. The temperature of the source was set at 220 °C. The mass spectrometer was operating in the electron impact (EI) mode at 70 eV. Gas chromatographic separations were operated on a HP-5MS column (30 m × 0.25 mm × 0.25 μm film thickness) with a constant He flow of 1.5 mL/min and a temperature gradient of 40 °C for 2 min, then 10 °C/min until 100 °C, then 4 °C/min up to 320 °C, hold time for 60 min. GC–MS interface was set at 320 °C. Mass spectra were produced in full detection mode over 70–800 amu. The peak assignment was based on the interpretation of mass spectra obtained with the OpenLab software and comparison with spectra available in the literature and NIST library 2.0. The same procedure (extraction, purification, silylation, GC–MS analysis) was applied to the Königsaue samples and the experimental birch tar samples.

### Micro-computed tomography (CT)

CT scans were recorded with the Phoenix v-tome-x s scanner (General Electric, Frankfurt am Main) of the Paleoanthropology High-Resolution CT Laboratory, Tübingen, selecting a resolution of about 4.7 microns. The reconstructed volumetric data (.vol) was sliced and the ISO surface of the pieces generated, using the Avizo Lite software.

## Results

### Chemical analysis

**Fig. 2:**
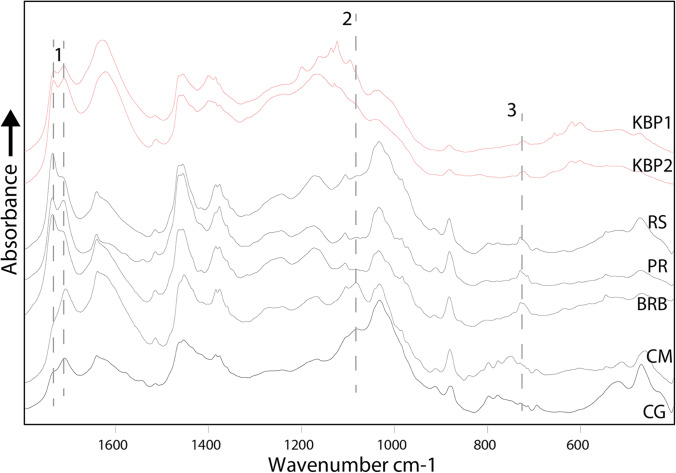
Infrared spectra of Königsaue birch tar. Averaged absorbance spectra obtained by transmission analysis (KBr pellets containing 0.7 mg of sample) of experimental birch tar samples compared with spectra recorded from the two Königsaue samples. KBP1 and 2, Königsaue artefacts; RS, raised structure; PR, pit roll; BRB, bark-roll buried; CM, condensation method; CG, cobble-groove. Broken lines show the regions discussed in the text. 1, double band caused by C = O in suberin (1735 cm^−1^) and oxidised triterpenoid bio- and degradation markers (1710 cm^−1^); 2, main Si–O-Si band of quartz at 1084 cm^−1^; 3, 727 cm^−1^ band caused by suberin

**Fig. 3:**
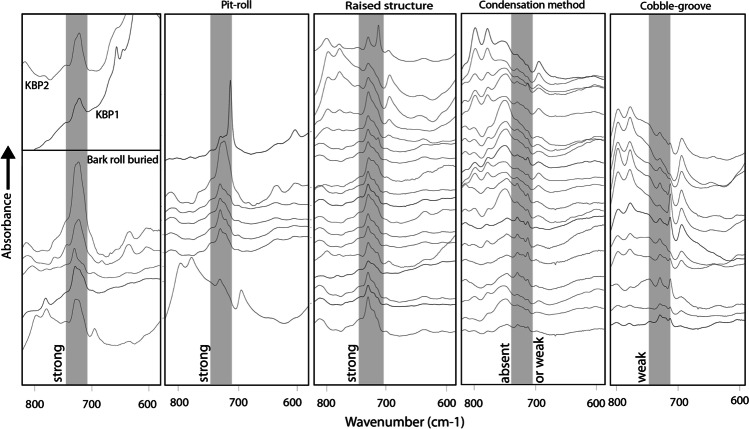
Infrared spectra of the region between 800 and 600 cm^−1^, showing the specific absorption band caused by suberin (marked by the grey bar) in 61 reference samples, compared to the Königsaue artefacts (top left). A sharp band at 713 cm^−1^ occurs beside the suberin band in some of the reference spectra. The band is caused by calcite impurities in the samples, most likely due to ash contaminants

In a first step, we analysed the 61 reference birch tar samples by transmission infrared (IR) spectroscopy (KBr pelleting) along with the two Königsaue tar artefacts. To investigate the samples’ chemistry, we averaged all IR spectra acquired on samples produced with the same techniques to obtain a representative tar spectrum for each technique (Fig. 2). These averaged spectra allow appreciating the spectral difference between different production methods, removing noise due to contaminations that only occur in a single spectrum (e.g. calcite from contamination with ash). The fingerprint regions of these spectra were compared with each other and with spectra acquired on the Königsaue artefacts. Noticeable differences in the averaged spectra of the five experimental production techniques are restricted to a few regions of their infrared spectra. The major difference is the inversion of the 1735 cm^−1^ and 1710 cm^−1^ double band in samples produced aboveground (condensation method and cobble-groove) as opposed to samples made belowground (pit-roll, bark roll buried, raised structure) (Fig. 2). The band at 1735 cm^−1^ is caused by C = O in suberin (Cordeiro et al. 1998), the bark’s polyester biopolymer epidermis that accounts for up to 6% of birch bark. While the major band of suberin in the fingerprint region lies at 1735 cm^−1^ (Miranda et al. 2013), the band’s presence in birch tar does not exclude the simultaneous presence of other esters that also cause absorptions at these wavenumbers. The 1710 cm^−1^ band is caused by C = O in different acids and aldehydes, amongst which (and that are most relevant to our samples) are oxidised biomarkers oleanolic acid and betulinic acid (Cîntă-Pînzaru et al. 2012) and their degradation markers. It is also present in degradation markers produced by the oxidation of biomarkers betulin and lupeol (i.e. betulone, lupenone) (Dwivedi et al. 2012). The band inversion thus reveals different concentrations of oxidised bio- and degradation markers in relation with the suberin content of the tars. In this sense, the Königsaue artefacts behave as experimental tars produced with the pit roll technique, both C = O bands having approximately equal heights. Experimental techniques are further set apart by a band at 1084 cm^−1^ that is only present in tars produced aboveground. The band is caused by the Si–O-Si stretching vibration of quartz (Farmer 1974). It is likely present in our samples because minor quartz impurities entered the tar when it was scraped from stone surfaces with a flint tool. The quartz band appears to be a proxy for tar-making techniques that rely on condensation and subsequent scraping. The band is absent in both Königsaue artefacts. However, in general terms, the presence of such a quartz band for identifying aboveground birch tar production methods might be limited, as tar may be contaminated with quartz impurities after its production. The third obvious difference between experimental samples is that tars made with the three underground techniques contain a band at 727 cm^−1^ that is absent or only very weak in tar made with aboveground techniques (Fig. 3). The band is caused by C-H deformation in aliphatic chains (Chen et al. 2022; Vahur et al. 2011) and is caused by the suberin fraction (Rocha et al. 2001) of the bark or long-chain fatty acids bound in the polyester biopolymer. Aboveground techniques, where tar is evaporated from the bark and then condensed above the bark itself, did not trigger significant transport of suberin into the tar in our experiments. A high suberin content appears to be a proxy for techniques involving underground processes where tar drips downwards (there is no exception to this in any of the 61 reference samples’ spectra: the suberin band is absent or very weak in condensation method tar, very weak in all cobble-groove tars and significantly stronger in tar made underground; Fig. 3). This interpretation is strengthened by the main suberin band at 1735 cm^−1^ that is present as shoulder only in aboveground techniques. The 727 cm^−1^ suberin band is present in both Königsaue artefacts. Thus, the spectral signature of the artefacts is most consistent with our reference tar samples made with one of the three underground techniques.

**Fig. 4:**
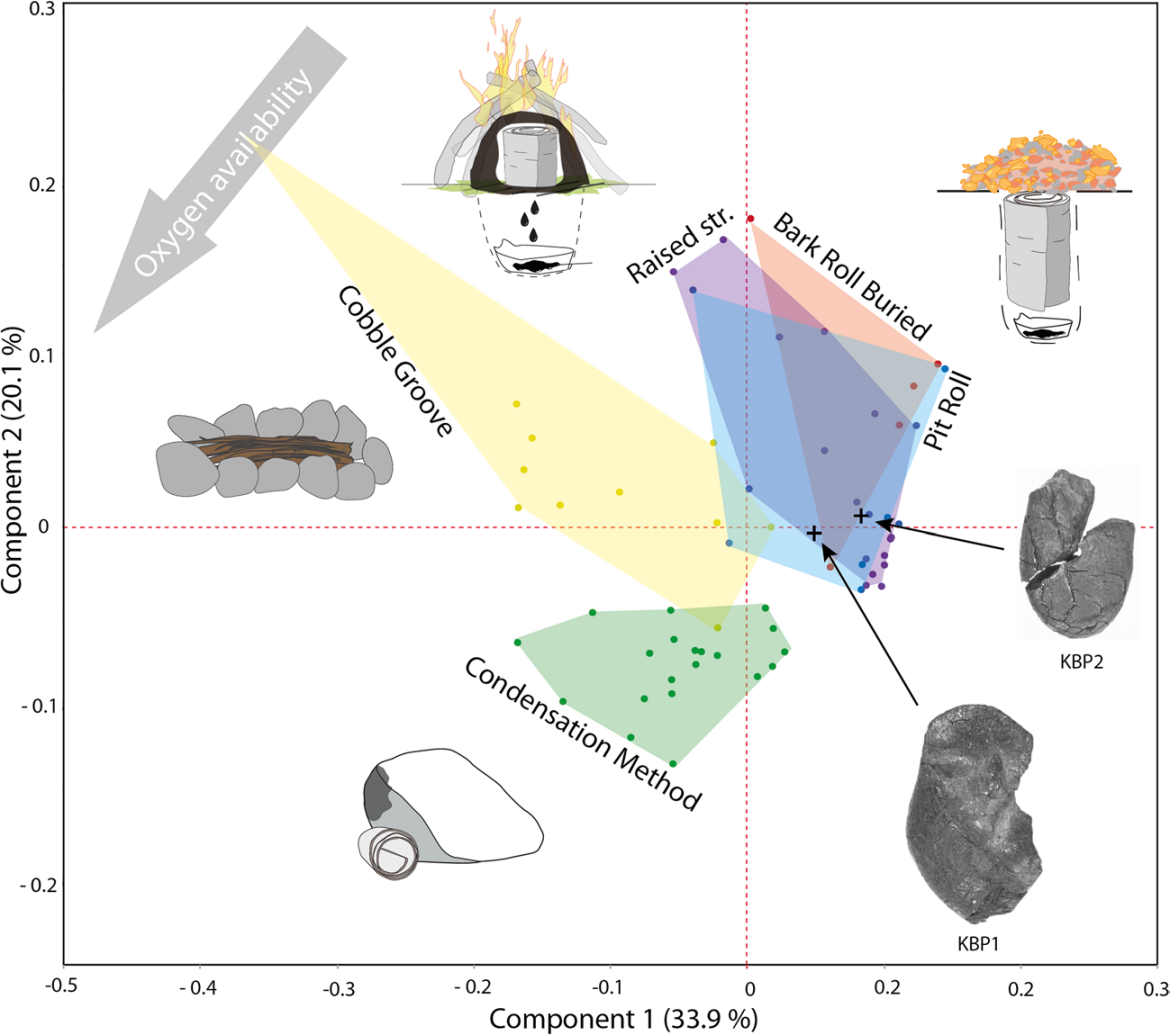
Principal component analysis (PCA) plot of first derivative data of infrared spectra recorded between 1800 and 400 cm^−1^. Note that the separation follows the predicted degree of oxygen availability during tar formation

There are however other minor spectral differences for which interpretation, in terms of the underlying molecular differences, is not straightforward. Some spectra contain weak supplementary bands that are absent in others. To use this chemical information concealed in the IR spectra of our samples, we conducted principal component analysis (PCA) on our spectral data. PCA of IR spectral data has been used to distinguish between birch tar and other adhesives (Chen et al. 2022). We amend this technique by using first derivative data calculated from our spectra, representing the slope on the original spectrum. These data are largely independent of variances in band height (differences in band height may be caused by impurities in unknown mixtures and background effects). Our PCA thus allows us to make statements on the similarities and dissimilarities of the infrared spectra of different samples, with regard to the presence/absence of absorption bands. The plot of the first two principal components (Fig. 4, but also see Fig. S7) separates the three underground techniques from the two aboveground methods. Tar made with the condensation method lies at one extreme of the plot, while tar made with the raised structure at the other. Separation appears to follow the predicted degree of oxygen availability during tar formation. The two Königsaue artefacts plot with the underground techniques, thus their spectrum is more similar to tar made belowground in low-oxygen conditions.

**Fig. 5:**
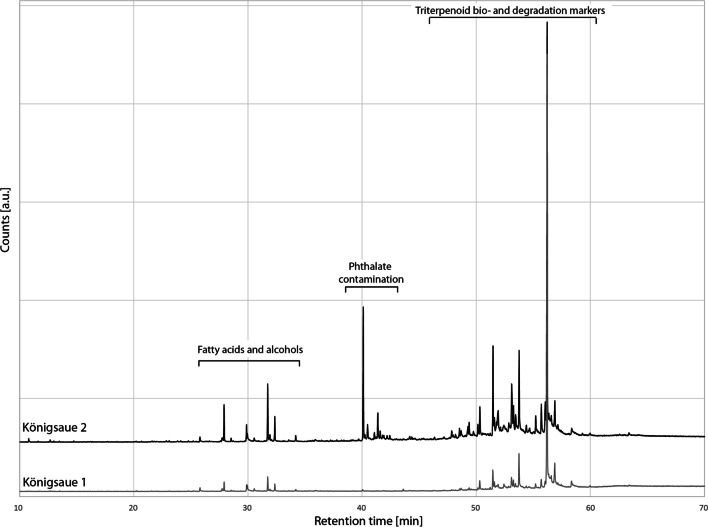
Complete chromatograms of the two Königsaue artefacts. Note that the phthalate contamination is only present in the younger Königsaue 2 piece. Both chromatograms contain fatty acids and triterpenoid bio- and degradation markers. Descriptions of these two regions can be found in the main text

**Fig. 6:**
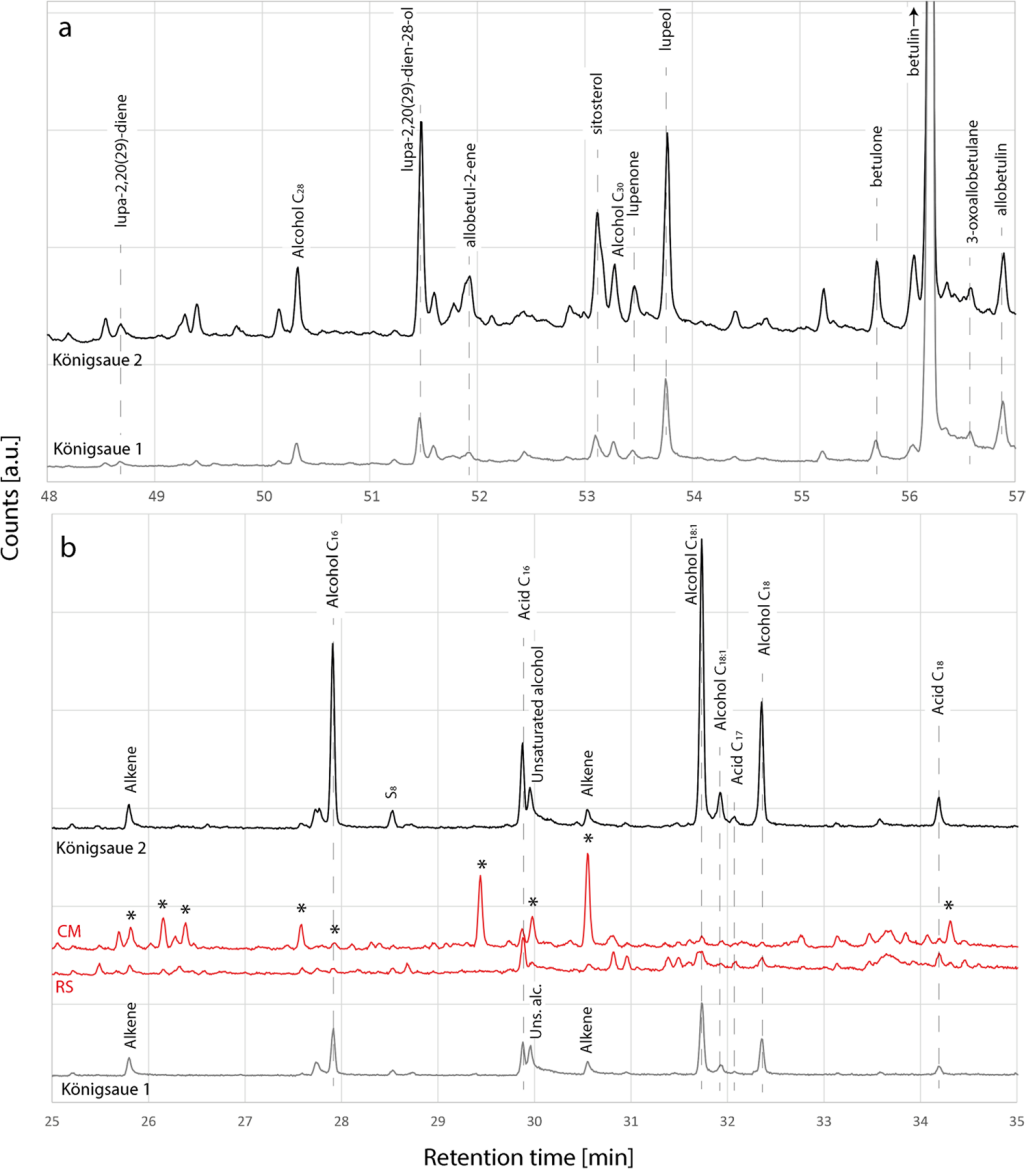
Partial chromatograms of the triterpenoid profile between 48 and 57 min (**a**) and of the acid profile between 25 and 35 min (**b**) of the two Königsaue birch tar artefacts. The acid profile in **b** is compared with the one of the reference tars made with the aboveground condensation method (CM) and the underground raised structure (RS). *, polycyclic aromatic hydrocarbons

To support our IR data, we conducted gas chromatography–mass spectrometry (GC–MS) analysis on the two Königsaue artefacts and on two randomly chosen experimental samples made with the condensation method and the raised structure. The chromatograms of the two Königsaue artefacts (Figs. 5 and 6) contain the typical peaks of triterpenoid bio- and degradation markers, confirming previous identifications as birch tar (Grünberg et al. 1999; Koller et al. 2001). The most abundant biomarker in both samples is betulin, although lupeol is also present. The most abundant degradation markers are lupa-2,20(29)-dien-28-ol, allobetulin, allobetul-2-ene and, in accordance with our IR spectra, both oxidised degradation markers betulone and lupenone. The younger Königsaue 2 contains an important contamination of phthalates (Fig. 5) that has previously been noticed (Koller et al. 2001) and the origin of which remains uncertain. One approach using GC–MS for understanding birch tar production methods is based on identifying a combination of different biomarkers in the linear and triterpenic acid regions of the tars’ chromatograms (Rageot et al. 2019). It has been proposed that the presence of even-numbered fatty acids C_16_ to C_22_, together with triterpenic acids and alcohols (in particular β-amyrin and oleanolic acid), odd-numbered fatty acids (e.g. C_21:0_) and diacids, is a proxy for double-pot distillation in later periods where tar making relies on using ceramics (Rageot et al. 2019). If this were applicable to aceramic tar making, it might be possible to separate raised structure birch tar from other tars on this basis. Both Königsaue artefacts contain fatty acids and alcohols C_16_, C_18_ and C_18:1_ (Fig. 6b). Both experimental tars, raised structure and condensation method, contain only traces of fatty acids C_16_ and C_18_ but none of the others present in the artefacts; no diacids were detected. Behenic acid C_22_, proposed to be the most characteristic fatty acid for identifying double-pot distillation when found in association with fatty acid C_21:0_ and diacids C_21_ and C_22_ (Rageot et al. 2019), is absent in Königsaue artefacts and reference samples. It therefore does not appear that the presence of fatty acids in the Königsaue birch tar is indicative of an underground production method (i.e. reference tars do not contain amounts of fatty acids similar to the artefacts). The most parsimonious explanation of fatty acids and alcohols in the two Königsaue artefacts is therefore that they result from soil contamination (Jambrina-Enríquez et al. 2019; Read et al. 2003) and cannot be used to make statements on the production technique (soil contamination is also supported by the presence of fatty alcohols C_28_ and C_30_ that are frequently derived from plant roots (Li et al. 2007), Fig. 6a). The chromatogram of reference tar made with the condensation method contains polycyclic aromatic hydrocarbons of different families (including di, tri- and tetra-aromatics). Such polyaromatic hydrocarbons are formed during incomplete combustion in wood/bark fires (Karp et al. 2020) and are common in soot (Avagyan et al. 2016). We therefore propose that their presence in birch tar may be a proxy for recognising aboveground production methods, based on condensation, where soot is incorporated in the tar. However, future studies should shed light on the stability of polyaromatic hydrocarbons over archaeological time periods before they may be understood as a potential proxy for tar production methods. Our raised structure reference tar and both Königsaue chromatograms are free from polyaromatic hydrocarbons. Although the chromatograms of the two artefacts show peaks at similar retention times, they are not caused by polyaromatics (Fig. 6b). Thus, the chromatographic signature of the two artefacts can be best explained by one of the belowground production techniques.

### The structure of the Königsaue birch tar

**Fig. 7:**
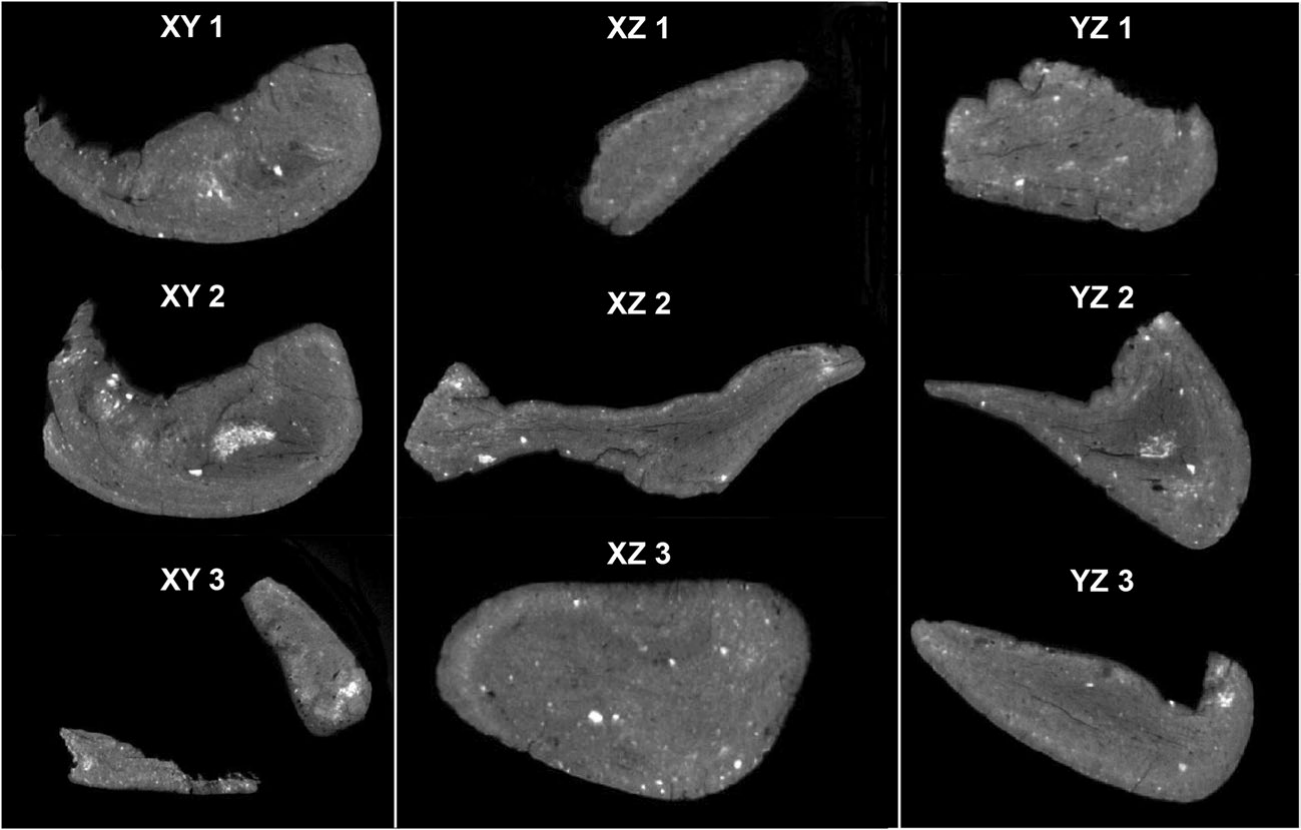
Three equidistant microCT slices of Königsaue 1, for each of the three axes of visualization (i.e., XY, XZ and YZ). The inclusions in Königsaue 1 appear to be small, rounded and about 2.15 times denser than the surrounding tar. They are likely sand inclusions

**Fig. 8:**
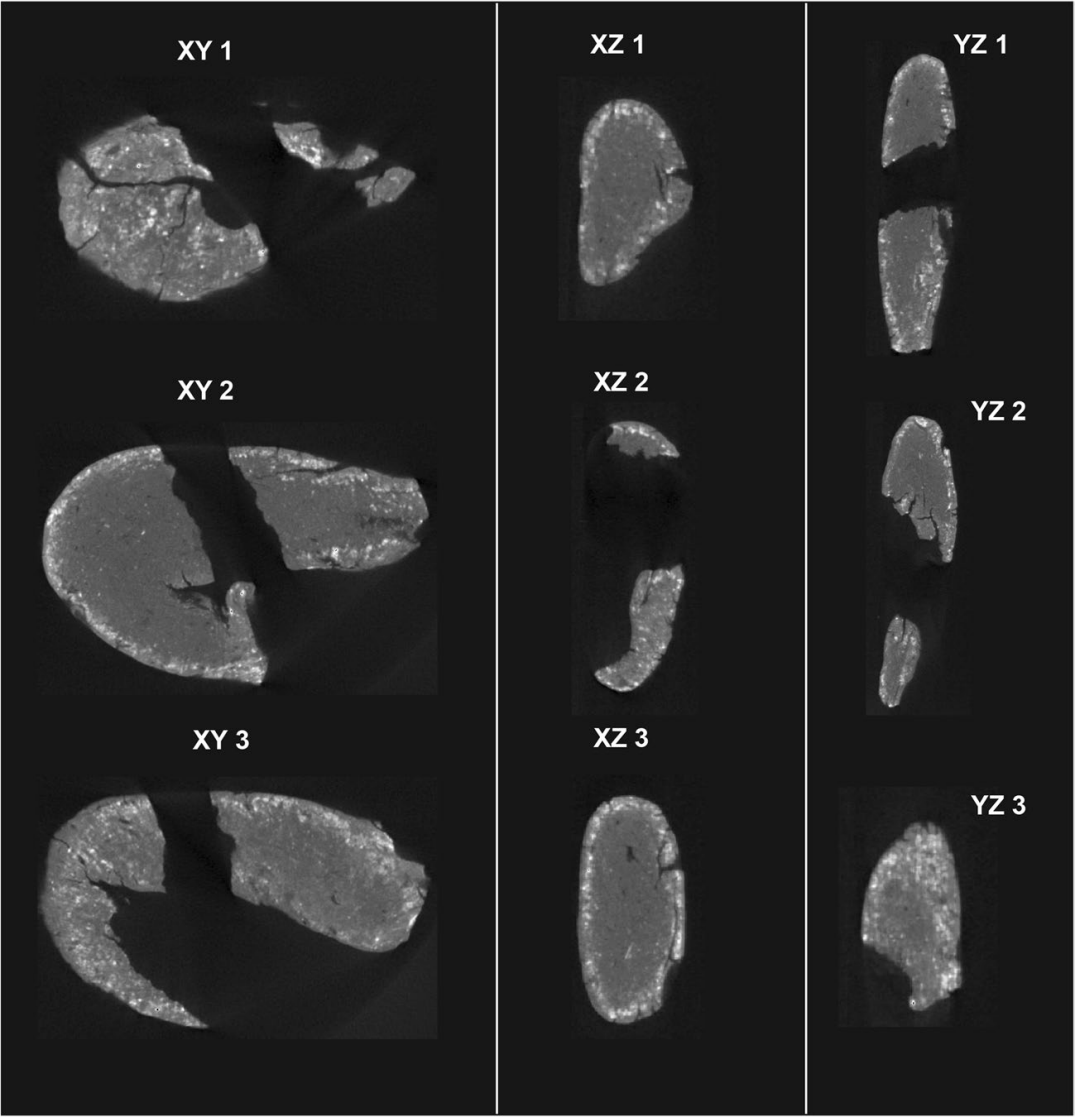
Three equidistant MicroCT slices of Königsaure 2, for each of the three axes of visualization (i.e., XY, XZ and YZ). Königsaue 2 shows a denser outer crust than Königsaue 1, which is most likely due to taphonomy, but no sand inclusions

To gain further insight into the structure of the two Königsaue artefacts, we recorded microCT scans. Both pieces are similar in overall density (1.23 g/cm^3^ and 1.18 g/cm^3^ for Königsaue 1 and 2 respectively, as calculated from a total volume of 1.103 cm^3^ and 0.705 cm^3^ and 1.35 g and 0.83 g). The larger Königsaue 1 piece shows signs of folding around the negative left by the stone tool it was attached to (Figs. 7 and 8). Bright inclusions with sizes between 0.2 and 0.6 mm and apparently rounded edges appear throughout the tar. Their grey value is 2.15 times higher than that of the surrounding tar. Assuming a roughly linear relationship between grey values and density in our CT scans (Mull 1984; Razi et al. 2014), the inclusions likely have a density of ~ 2.58 g/cm^3^, a value reasonably close to minerals quartz and feldspar. It therefore appears that these inclusions comprise fine sand grains incorporated in the tar. This sediment contamination accounts for 0.5% of the total volume of the piece (5 mm^3^). The smaller Königsaue 2 artefact does not contain such inclusions and its structure appears more homogeneous (no folding). Its outer zone has a bright cloudy aspect parallel to the object’s surface (Fig. 8). This may be caused by the taphonomic take-up of minerals that are denser than the tar itself. Thus, the two birch tar artefacts differ in that one is more contaminated and apparently more kneaded than the other. The sand grains in Königsaue 1 are likely too few and too separated to have the effect of a loading agent that might have been added to modify the strength of the tar (Zipkin et al. 2014). It is also uncertain if the soil contamination conceals information about the production technique. It may simply reveal that this piece was recycled more often than the younger Königsaue 2 artefact.

## Discussion and conclusion

The overall chemical signature of both Königsaue tar artefacts is most similar to tars made belowground. A question arising from this is if this similarity might be caused by post-depositional processes. We compare modern tar with artefacts older than 40 ka and processes, such as oxidation, may occur over prolonged time periods in soil. However, in our case, taphonomic factors can be ruled out because we identified suberin in the Königsaue pieces. Suberin is a polymer naturally forming in the bark of birch trees (Rocha et al. 2001) and not by post-depositional processes. If suberin is only included in birch tar made with underground techniques in low oxygen environments, its presence in the Königsaue tar is unambiguously pointing towards the use of one of these techniques at Königsaue.

Thus, both Königsaue artefacts seem to have been made with a method that involved a restriction of oxygen flow, for example in an underground structure. Some authors have described such techniques as more technically (Kozowyk et al. 2017) and cognitively complex (Wadley 2013) than others. While the concept of complexity as a direct reflection of advanced cognitive processes has been criticized (Tennie and Hedwig 2009), early pyrotechnology has been described as a good indicator of cultural diffusion (MacDonald et al. 2021). Our finding of an elaborate birch tar-making process therefore adds to previous arguments that Neanderthals were capable of complex expressions and cultural transmission (Hayden 1993; Lind et al. 2013; Roebroeks and Soressi 2016). Many of these arguments are based on comparisons of the material culture of Neanderthals and contemporary *Homo sapiens*. And indeed, both species practiced similar techniques and used similar tools. Bone tools (Soressi et al. 2013), personal ornaments (Rodríguez-Hidalgo et al. 2019) and ochre (Roebroeks et al. 2012), most likely used for symbolic expressions, are amongst them. Most of these manifestations appeared earlier in *Homo sapiens*, so claims of acculturation were brought forward to explain some of the Neanderthal artefacts (Hublin et al. 2020). Recent evidence indicates possible early *Homo sapiens* incursions into southern Europe, overlapping (France (Slimak et al. 2022)) or predating (Southern Greece (Harvati et al. 2019)) the time frame of the Königsaue birch tar artefacts. These findings are supported by paleo-genetic analyses (Peyrégne et al. 2022; Posth et al. 2017). Thus, the possibility of cultural exchange cannot be completely excluded and should be further investigated. However, given the great geographic and temporal expanse separating the Königsaue artefacts from those indications, we consider a local cultural evolution as the more parsimonious interpretation. Another argument against acculturation, in the sense of *Homo sapiens* showing Neanderthals how to make birch tar, is that, to date, there are no archaeological remains associated with Palaeolithic *Homo sapiens* sites that would have been chemically identified to be birch tar. If the Campitello dates of 200 ka are correct, Neanderthals made tar in the Middle Palaeolithic (Mazza et al. 2006), more than 100 ka before the earliest known instance of adhesive production in *Homo sapiens* (Charrié-Duhaut et al. 2013). Thus, European birch tar may be one of the best proxies for independent cultural processes in Neanderthals. However, birch tar in general may be produced with a cognitively undemanding technique (although this would not necessarily indicate the absence of innovation, see refs. (Schmidt et al. 2022, 2023)), or even be the result of unintentional processes in open-air fires (Schmidt et al. 2019). What our study suggests is that, at least in the end of the presence of Neanderthals in Europe, this was not the case. Underground transformative techniques, like those used to make the Königsaue artefacts, are more difficult than aboveground techniques because some elements cannot be observed or corrected after the procedure began (Schmidt 2021; Stolarczyk and Schmidt 2018). It also appears unlikely that Neanderthals fully understood these invisible elements. Incomprehensible processes have been called *cognitively opaque knowledge* (Csibra and Gergely 2011), and might be a strong indicator of cultural transmission in Neanderthals. However, the implications of underground tar making may even go beyond documenting cultural transmission and social learning. Because of the higher likelihood of failure when performing underground techniques (Blessing and Schmidt 2021), specific recipes must be followed and copied precisely. Such high-fidelity copying has been argued to be a key element of cumulative culture (Lewis and Laland 2012). While this is not unanimously accepted (Saldana et al. 2019), the underground production of tar unambiguously documents a *ratcheting effect* indicative of cumulative culture (Tomasello 2009). This is so because Neanderthals could not likely invent such a technique ex nihilo. Aboveground techniques are more likely to be the result of fortuitous discoveries (Schmidt et al. 2019). Underground tar making was more likely a technical improvement based on previous, simpler techniques (ratcheting). This is also true for underground techniques that have been described as being simpler, such as the pit-roll technique (Kozowyk et al. 2017), because they also involve processes that cannot be directly observed. As all underground processes we experimented with contain invisible (cognitively opaque (Csibra and Gergely 2011)) processes, information must be conveyed orally or by other social learning mechanisms. A shift of tar-making technology from aboveground to underground techniques satisfies three of the core criteria proposed to be minimum requirements for a population to exhibit cumulative cultural evolution (Mesoudi and Thornton; Tomasello 2009): it is (i) a change in a behaviour that must be (ii) transferred via social learning and that (iii) led to an improvement in performance (i.e. underground tar making is more efficient). The fourth criterion proposed as core, the repetition of steps (i) to (iii) to generate sequential improvement over time, cannot be investigated unambiguously based on the few known Neanderthal tar artefacts. Our interpretation that the Königsaue tar represents cumulative cultural evolution is further strengthened by the fact that it was produced towards the end of the Neanderthal occupation in Europe. Thus, what we show here for the first time is that Neanderthals invented and refined a transformative technique, most likely independently of the influence from *Homo sapiens*. This might be supported by analyses of older tar fragments attributed to Neanderthals (the two Campitello artefacts from example), providing an exciting prospect for future research.

There are only a few other transformative techniques that may be understood to document cultural evolution to a similar degree. Heat treatment of stone for tool knapping is amongst them. While stone heat treatment in Africa predates the Königsaue birch tar artefacts (Schmidt et al. 2020), it has been shown that in South Africa, it did not involve invisible underground processes (Schmidt et al. 2015). Thus, if the Campitello dates of ~ 200 ka are correct, Neanderthal birch tar making seems to be the first documented manifestation of this kind in human evolution.

## Supplementary information

Below is the link to the electronic supplementary material.

Supplementary file1 (PDF 2089 KB)

## Data Availability

All data needed to evaluate the conclusions in the paper are present in the paper and/or the Supplementary Information.
