# Supplementary material for: Production method of the Königsaue birch tar documents cumulative culture in Neanderthals
Source: Archaeol Anthropol Sci. 2023 May 22;15(6):84. doi: 10.1007/s12520-023-01789-2 (PMC10202989; doi:10.1007/s12520-023-01789-2)
Supplement: Supplementary file 1 — Supplementary file1 (PDF 2089 KB) [file 12520_2023_1789_MOESM1_ESM.pdf]

Supplementary information for:

Production method of the Königsaue birch tar documents cumulative culture in Neanderthals

Patrick Schmidt, Tabea J. Koch, Matthias A. Blessing, F. Alexandros Karakostis, Katerina Harvati, Veit Dresely, Armelle Charrié-Duhaut

Corresponding author: Patrick Schmidt, patrick.schmidt@uni-tuebingen.de

This PDF file includes:

Supplementary Text
Figs. S1 to S11
Tables S1 to S3

Supplementary Text

The controversy surrounding the dating of Königsaue birch tar

The relative chronology of the two Königsaue tar pieces is unambiguous because the stratigraphic integrity of the site is unquestioned. Thus, we know that one piece is older than the other one, but not by how much and neither how old these pieces are.

The absolute age of the Paleolithic horizons of Königsaue has been widely debated (Kurzweil and Todtenhaupt 1992; Koller, Baumer, and Mania 2001; Ruebens 2013; Sykes 2015; Mania 2015; Wiśniewski 2014; Rots 2015). Reason for the controversy is the discrepancy between the radiocarbon dates taken from the tar pieces themselves and the geochronological assessment of the stratigraphic sequence where the tar pieces originate from.

Mania and Toepfer (Mania and Toepfer 1973) attributed the Middle Paleolithic assemblages that accompanied the birch tar pieces to the Brörup interstadial in MIS 5c based on the geochronology of the lake sediments, an age estimate that has since been defended by Mania (Mania 2015, 1999; Mania 2006). Additionally, a range of 1000 years between horizon A and C has been assumed based on the suggested length of the Brörup interstadial (Mania and Toepfer 1973). This is the origin of the 80 ka date associated with the Königsaue tar pieces. More recent research placed the Brörup interstadial around the time of 100 ka, however, and today, the Odderade interstadial would coincide with an age of 80 ka (Berner and Streif 2000). The original geostratigraphic dating was partly based on faunal remains associated with warmer conditions (Mania and Toepfer 1973), so a final decision to which interstadial they might belong is not possible.

There are currently three radiocarbon dates associated with the Königsaue tar – two directly taken from the pieces themselves and one on a bone from horizon A. The sample from the tar piece Königsaue A yielded an age of 50.6 to 41.4 ka calBP (Grünberg et al. 1999). Trying to recalibrate the age for Königsaue B (48.4 ± 3.7 ka uncalBP) using OxCal 4.4 on the IntCal20 curve yielded an invalid age, meaning that the original age is most likely infinite. Given the time of when these dates were obtained, it seems likely that both should actually be treated as infinite ages, which is essentially Mania's position (Mania 2015). Yet, a recent AMS age of a bone from horizon A yielded an age of 45.9 to 44.5 ka calBP (Picin 2016) which would fall within the range of the tar piece Königsaue A. This is all the more puzzling as horizon A is the lowermost archaeological layer and the infinite age comes from the younger horizon. Mania has argued that all three layers cover a time span of about 1000 years. On first glance, it therefore seems possible that the lowermost horizon is that young. However, Mania's assessment is built upon the geochronology, which means accepting the short time succession of the archaeological horizons automatically means rejecting the radiocarbon dates given the available evidence as of today.

Hence, while the archaeological context of the tar pieces from Königsaue is undoubtedly Middle Paleolithic, the debate about their age and the chronological relationship between the pieces is far from settled. Based on the current evidence and (most likely failed) attempts to date the pieces using radiocarbon, one possible conclusion is that the pieces are older than the range of radiocarbon dating. We follow Picin's notion that an extensive dating program is necessary to resolve this problem (Picin 2016), if it can be resolved at all.

A list of Middle Paleolithic adhesive finds from Europe and the Levant

Table S1. List of known adhesives attributed to Neanderthals.

Site	Country	Approx. age	Type	Reference
Inden Altdorf	DE	~120 Ka	Not yet analysed	Ref. (Pawlik and Thissen 2011)
Fossellone and Sant'Agostino caves	IT	40-55 ka	conifer resin (also mixed with beeswax)	Ref. (Degano et al. 2019)
Königsaue	DE	45-80 ka	Birch tar	Ref. (Grünberg et al. 1999)
Campitello	IT	~200 ka	Birch tar	Ref. (Mazza et al. 2006)
Zandmotor	NL	50 ka	Birch tar	Ref. (Niekus et al. 2019)
Umm el Tlel	SY	40-70 ka	Bitumen	Ref. (Boëda et al. 2008)
Hummal	SY	50-80 ka	Bitumen	Ref. (Hauck et al. 2013)

Precisions on the methods used for experimental birch tar production

The condensation method

First published in 2019 (Schmidt et al. 2019), the condensation method represents the simplest way of making birch tar we currently know. It is an open-air and readily observable method that only requires stones, birch bark and fire. A roll of birch bark is placed beneath a subparallel stone surface and lit. While the bark is burning only minor adjustments need to be made as the fire progresses. Tar condenses on the stone surface and can be scraped off using a flake or similar tool. The process can be repeated until the desired amount of birch tar has been produced. It is possible for one person to operate multiple stones at the same time, but three stones were found to be the best compromise between manageability and output rate.

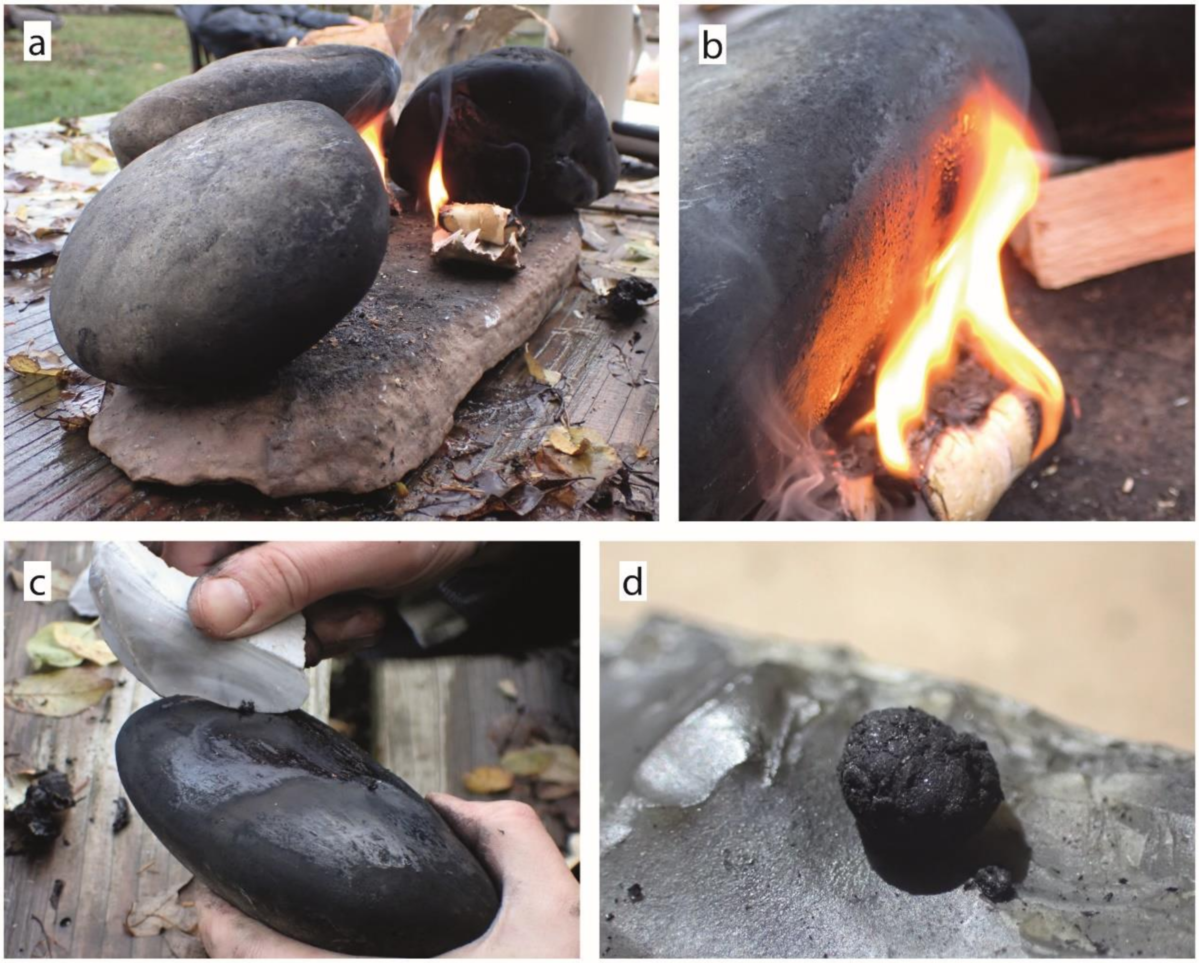

Figure S1. Experimental setup of the condensation method. (a) Three cobbles are operated simultaneously. (b) During the experiment, tar condenses onto the tilted cobble that can (c) be scraped off using a flint flake. (d) The amount of tar collected during one experimental run.

The cobble-groove technique

This method describes a variation of the condensation method (Koch and Schmidt 2022). It is a semi-open-air method that takes place aboveground with restricted air in-flow. For the setup, an approximately 30cm long groove is dug using a wooden stick. Flat and smooth river cobbles are placed at the bottom of this groove and further stones are placed upright on each side. The structure is filled with birch strips of a similar length and width as to fit into the groove. This bark-filled structure is covered with additional cobbles, leaving just one opening at the extremity of the structure. The gaps between the side and top cobbles are filled with wetted sediment. At the opening, the bark is lit. Depending on the quantity of bark used, the flames extinguish after 15-30 minutes. In most cases, no additional attention is required. However, when the flames threaten to go out, air needs to be blown into the opening to keep the bark burning. When the bark strips are completely charred, tar that had condensed onto the top and side cobbles can be scraped off using flint flakes.

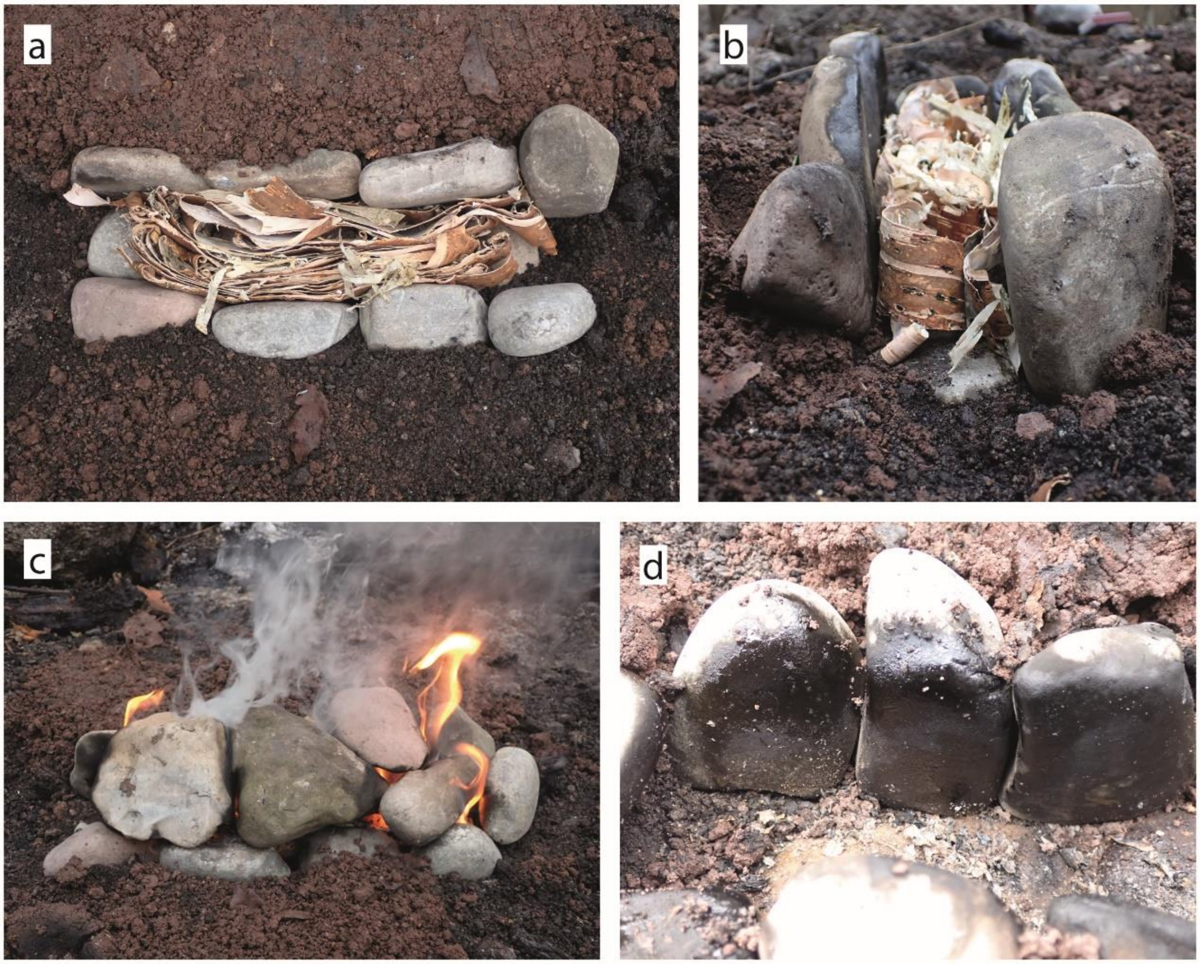

Figure S2. Experimental setup of the cobble-groove condensation method. (a-b) The groove filled with strips of birch bark. (c) The structure after lighting of the bark. (d) Tar condensed onto the side cobbles.

The pit roll technique

With this method, the reaction to make birch tar happens underground and out of sight of the operator. In our own experiments we also found it to be the most unreliable method of production. A small pit needs to be dug into the ground, just big enough to hold a roll of birch bark. Although the technique is sometimes described (see for example ref. (Kozowyk et al. 2017)) to derive from descriptions in ref. (Pawlik 2004), where the bark roll would be set on fire and then put into the hole, we found no such description in ref. (Pawlik 2004). Regardless, lighting the bark roll itself could not be reproduced and an external source of heat had to be added (Kozowyk et al. 2017). Glowing embers on top of the pit with the bark roll inside provided this external heat source in our experiments. The tar drops into a receptacle placed at the bottom of the pit and can be collected from there. However, in our experience, the tar is mostly trapped within the layers of the bark roll, if it forms at all. The pit roll technique seems to be difficult to control, the tar output is minimal and the method is not consistently successful.

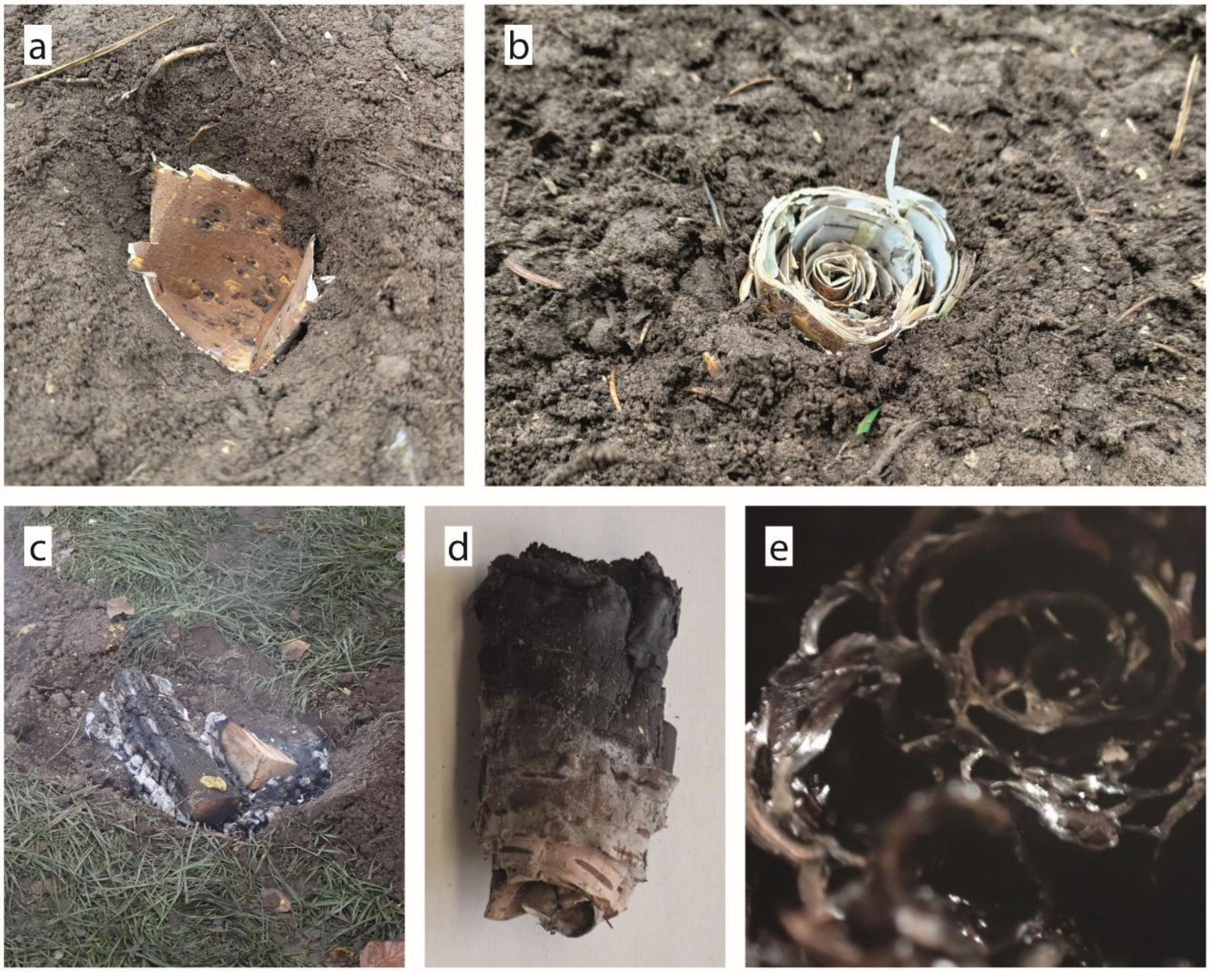

Figure S3. Experimental setup of the pit roll method. (a) The pit with a birch bark receptacle. (b) The bark roll before the experiment. (c) Ambers cover the buried roll. (d) A bark roll that did not char completely. (e) Close up image of tar that remained in the bark roll.

The bark roll buried technique

A roll of birch bark is placed lying horizontally in a pit of similar length. The roll is buried deep enough as to be covered with ~0.5 - 1 cm of sediment. A fire is lit on top of the buried bark roll. After approximately 30 minutes, the embers can be removed and the bark roll can be excavated. During the process, the bark chars and tar forms within the layers of the roll. Because the roll is placed in a horizontal position, only small amounts of tar are lost in the surrounding sediment. However, the remaining tar adheres to the charred bark roll and is difficult to collect.

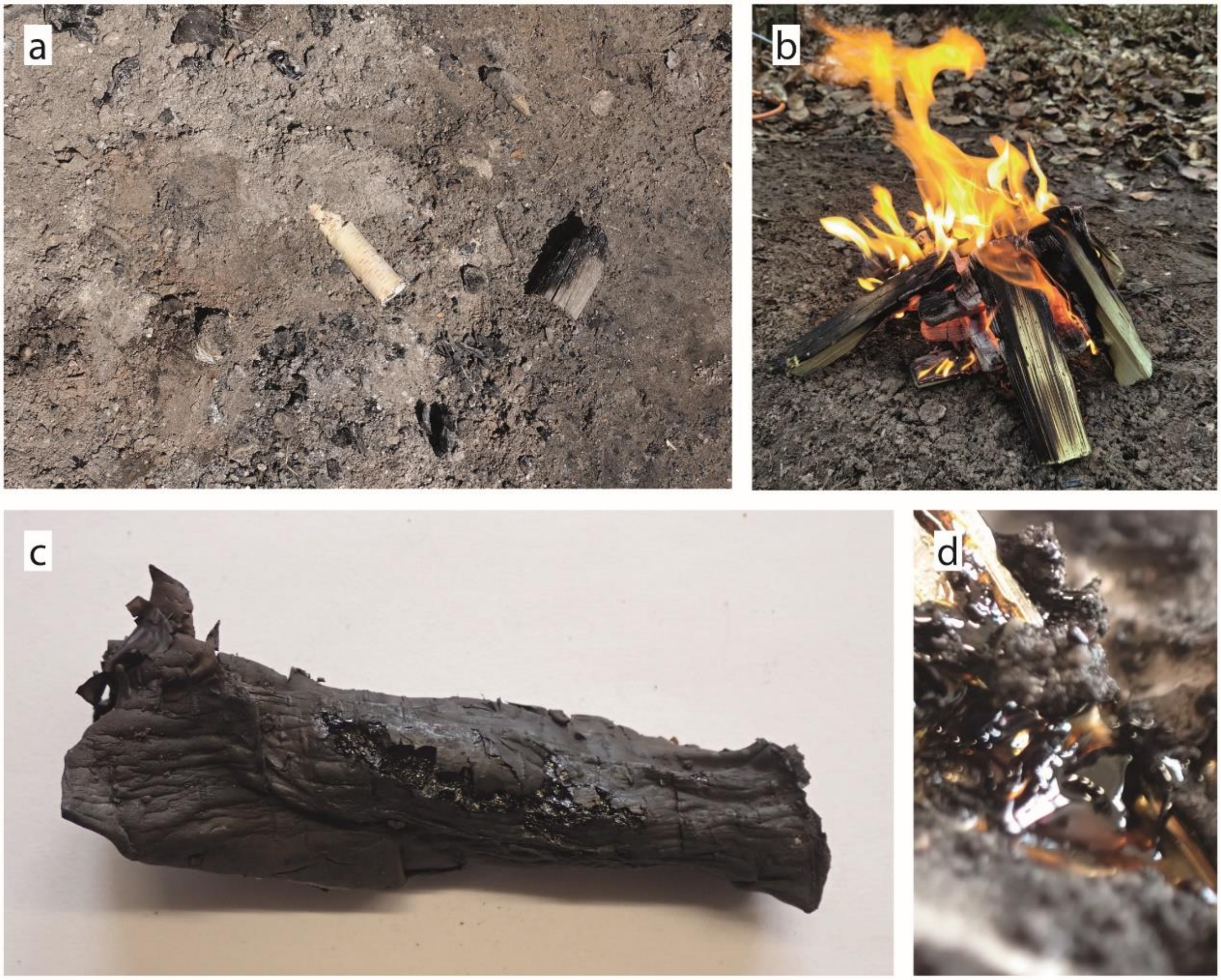

Figure S4. Experimental setup of the buried bark roll method. (a) Top view of a bark roll. (b) The fire burns directly above the buried roll. (c) The bark roll completely charred during this experiment. (d) Close up image of the tar that adheres to the charred remains of the bark.

The raised structure

The raised structure could be considered an aceramic version of the historically known double-pot distillation using ceramic containers. In comparison to the other aceramic tar making techniques, the so-called raised structure requires the most steps to be carried out (Kozowyk et al. 2017). First, a receptacle (e. g. made from birch bark) is placed in a small pit. This pit (lower chamber) is then covered with a grit made of thin twigs. A previously made birch roll is placed onto the grit and covered with an earthen dome (upper chamber) made of sediment and clay. A fire is lit around the structure. This technique requires a certain amount of time and effort, as well as skill in terms of temperature control. The bark roll chars and tar drips into the receptacle. After the fire has burnt out (2-3 hours), the dome can be opened, and the tar collected. The structure can also be left to cool down (e. g. overnight).

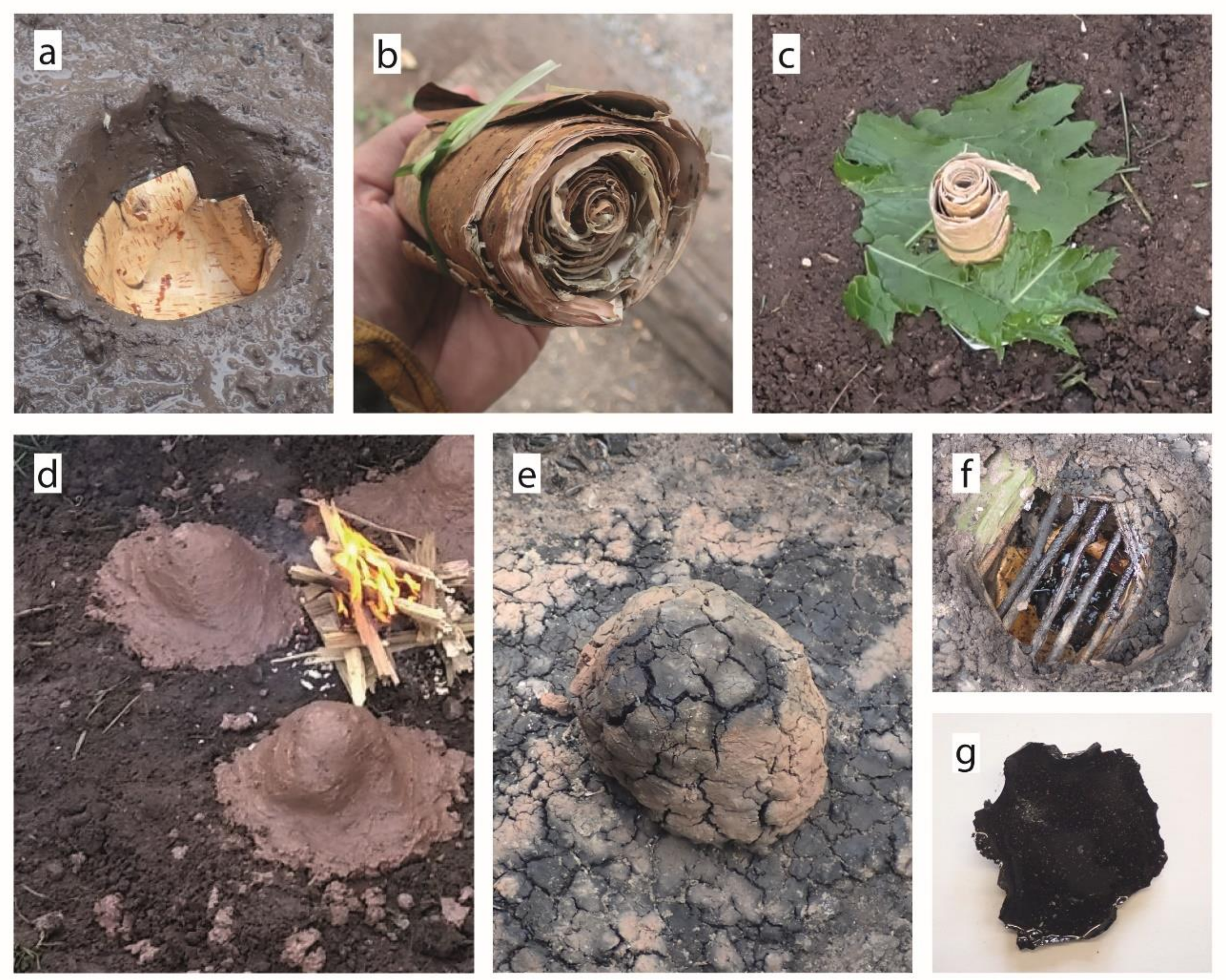

Figure S5. Experimental setup of the raised structure. (a) A bark receptacle placed at the bottom of the pit. (b) A tightly rolled bark roll to be placed onto the grit. (c) Fresh leaves are placed around to roll to prevent sediment falling down. (d) Two raised structures made of clay and sediment when lighting the fire. (e) Cracks in the earthen mound are visible after the firing. (f) Tar that dripped into the bark receptacle in the lower chamber below the grit. (g) Solidified tar after leaving a raised structure cool down over night.

List of experimental reference samples

The birch bark used to produce the 61 reference samples was collected from several trees. The exact number of trees was not recorded but can be estimated to > 80. Bark was collected from the stem section of trees older than 15 years. Dead bark was collected from fallen trees lying on the ground in forests. Fresh bark was removed from freshly cut trees (trees were cut with permission of the Mayor’s office) that were felled the same day.

Table S2. Sample IDs, production methods, production dates, type of bark used and origin of the birch trees used to produce the 61 reference birch tar samples. BRB = Bark roll buried; CG = Cobble Groove; CM = Condensation method; PR = Pit roll; RS = raised structure.

Sample ID	Method	Date	Bark type	Species	Origin of the tree
2	BRB	05.07.2020	Dead Bark	*B. pendula*	Weil im Schönbuch, Baden-Württemberg, DE
3	BRB	05.07.2020	Dead Bark	*B. pendula*	Weil im Schönbuch, Baden-Württemberg, DE
25	BRB	21.11.2020	Fresh Bark	*B. pendula*	Bad Liebenzell, Baden-Württemberg, DE
26	BRB	21.11.2020	Fresh Bark	*B. pendula*	Bad Liebenzell, Baden-Württemberg, DE
27	BRB	21.11.2020	Fresh Bark	*B. pendula*	Bad Liebenzell, Baden-Württemberg, DE
37	CG	13.12.2020	Dead Bark	*B. pendula*	Weil im Schönbuch, Baden-Württemberg, DE
38	CG	13.12.2020	Dead Bark	*B. pendula*	Weil im Schönbuch, Baden-Württemberg, DE
39	CG	13.12.2020	Dead Bark	*B. pendula*	Weil im Schönbuch, Baden-Württemberg, DE
40	CG	13.12.2020	Dead Bark	*B. pendula*	Weil im Schönbuch, Baden-Württemberg, DE
41	CG	13.12.2020	Dead Bark	*B. pendula*	Weil im Schönbuch, Baden-Württemberg, DE
45	CG	11.09.2022	Dead Bark	*B. pendula*	Weil im Schönbuch, Baden-Württemberg, DE
46	CG	11.09.2022	Dead Bark	*B. pendula*	Weil im Schönbuch, Baden-Württemberg, DE
62	CG	03.11.2022	Dead Bark	*B. pendula*	Liège, BE
63	CG	03.11.2022	Dead Bark	*B. pendula*	Liège, BE
42	CG	10.01.2121	Dead Bark	*B. pendula*	Weil im Schönbuch, Baden-Württemberg, DE
43	CG	10.01.2121	Dead Bark	*B. pendula*	Weil im Schönbuch, Baden-Württemberg, DE
1.1	CM	28.07.2018	Dead Bark	*B. pendula*	Weißwasser, Saxony, DE
1.2	CM	19.07.2020	Dead Bark	*B. pendula*	Weißwasser, Saxony, DE
1	CM	05.07.2020	Dead Bark	*B. pendula*	Weil im Schönbuch, Baden-Württemberg, DE
7	CM	19.07.2020	Dead Bark	*B. pendula*	Weil im Schönbuch, Baden-Württemberg, DE
9	CM	03.08.2020	Dead Bark	*B. pendula*	Weil im Schönbuch, Baden-Württemberg, DE

10	CM	03.08.2020	Dead Bark	*B. pendula*	Weil im Schönbuch, Baden-Württemberg, DE
18	CM	15.11.2020	Dead Bark	*B. pendula*	Weil im Schönbuch, Baden-Württemberg, DE
19	CM	15.11.2020	Dead Bark	*B. pendula*	Weil im Schönbuch, Baden-Württemberg, DE
20	CM	15.11.2020	Dead Bark	*B. pendula*	Weil im Schönbuch, Baden-Württemberg, DE
32	CM	21.11.2020	Fresh Bark	*B. pendula*	Bad Liebenzell, Baden-Württemberg, DE
33	CM	21.11.2020	Fresh Bark	*B. pendula*	Bad Liebenzell, Baden-Württemberg, DE
34	CM	21.11.2020	Fresh Bark	*B. pendula*	Bad Liebenzell, Baden-Württemberg, DE
35	CM	21.11.2020	Fresh Bark	*B. pendula*	Bad Liebenzell, Baden-Württemberg, DE
47	CM	11.09.2022	Dead Bark	*B. pendula*	Weil im Schönbuch, Baden-Württemberg, DE
48	CM	02.11.2022	Dead Bark	*B. pendula*	Weil im Schönbuch, Baden-Württemberg, DE
55	CM	03.11.2022	Dead Bark	*B. pendula*	Liège, BE
60	CM	03.11.2022	Dead Bark	*B. pendula*	Liège, BE
61	CM	03.11.2022	Dead Bark	*B. pendula*	Liège, BE
64	CM	14.11.2022	Dead Bark	*B. pendula*	Weil im Schönbuch, Baden-Württemberg, DE
65	CM	14.11.2022	Dead Bark	*B. pendula*	Weil im Schönbuch, Baden-Württemberg, DE
11	PR	12.09.2020	Dead Bark	*B. pendula*	Weil im Schönbuch, Baden-Württemberg, DE
12	PR	12.09.2020	Dead Bark	*B. pendula*	Weil im Schönbuch, Baden-Württemberg, DE
13	PR	26.09.2020	Dead Bark	*B. pendula*	Weil im Schönbuch, Baden-Württemberg, DE
15	PR	26.09.2020	Dead Bark	*B. pendula*	Weil im Schönbuch, Baden-Württemberg, DE
29	PR	21.11.2020	Fresh Bark	*B. pendula*	Bad Liebenzell, Baden-Württemberg, DE
30	PR	21.11.2020	Fresh Bark	*B. pendula*	Bad Liebenzell, Baden-Württemberg, DE
36	PR	15.11.2020	Dead Bark	*B. pendula*	Weil im Schönbuch, Baden-Württemberg, DE

5	RS	05.07.2020	Dead Bark	*B. pendula*	Weil im Schönbuch, Baden-Württemberg, DE
16	RS	24.10.2020	Dead Bark	*B. pendula*	Weil im Schönbuch, Baden-Württemberg, DE
17	RS	24.10.2020	Dead Bark	*B. pendula*	Weil im Schönbuch, Baden-Württemberg, DE
21	RS	21.11.2020	Fresh Bark	*B. pendula*	Bad Liebenzell, Baden-Württemberg, DE
22	RS	21.11.2020	Fresh Bark	*B. pendula*	Bad Liebenzell, Baden-Württemberg, DE
23	RS	21.11.2020	Fresh Bark	*B. pendula*	Bad Liebenzell, Baden-Württemberg, DE
24	RS	21.11.2020	Fresh Bark	*B. pendula*	Bad Liebenzell, Baden-Württemberg, DE
31	RS	15.11.2020	Dead Bark	*B. pendula*	Weil im Schönbuch, Baden-Württemberg, DE
49	RS	02.11.2022	Dead Bark	*B. pendula*	Liège, BE
50	RS	02.11.2022	Dead Bark	*B. pendula*	Liège, BE
51	RS	02.11.2022	Dead Bark	*B. pendula*	Liège, BE
52	RS	02.11.2022	Dead Bark	*B. pendula*	Liège, BE
53	RS	02.11.2022	Dead Bark	*B. pendula*	Liège, BE
54	RS	02.11.2022	Dead Bark	*B. pendula*	Liège, BE
56	RS	02.11.2022	Dead Bark	*B. pendula*	Liège, BE
57	RS	02.11.2022	Dead Bark	*B. pendula*	Liège, BE
58	RS	02.11.2022	Dead Bark	*B. pendula*	Liège, BE
59	RS	02.11.2022	Dead Bark	*B. pendula*	Liège, BE

Supplementary information on Infrared spectroscopy

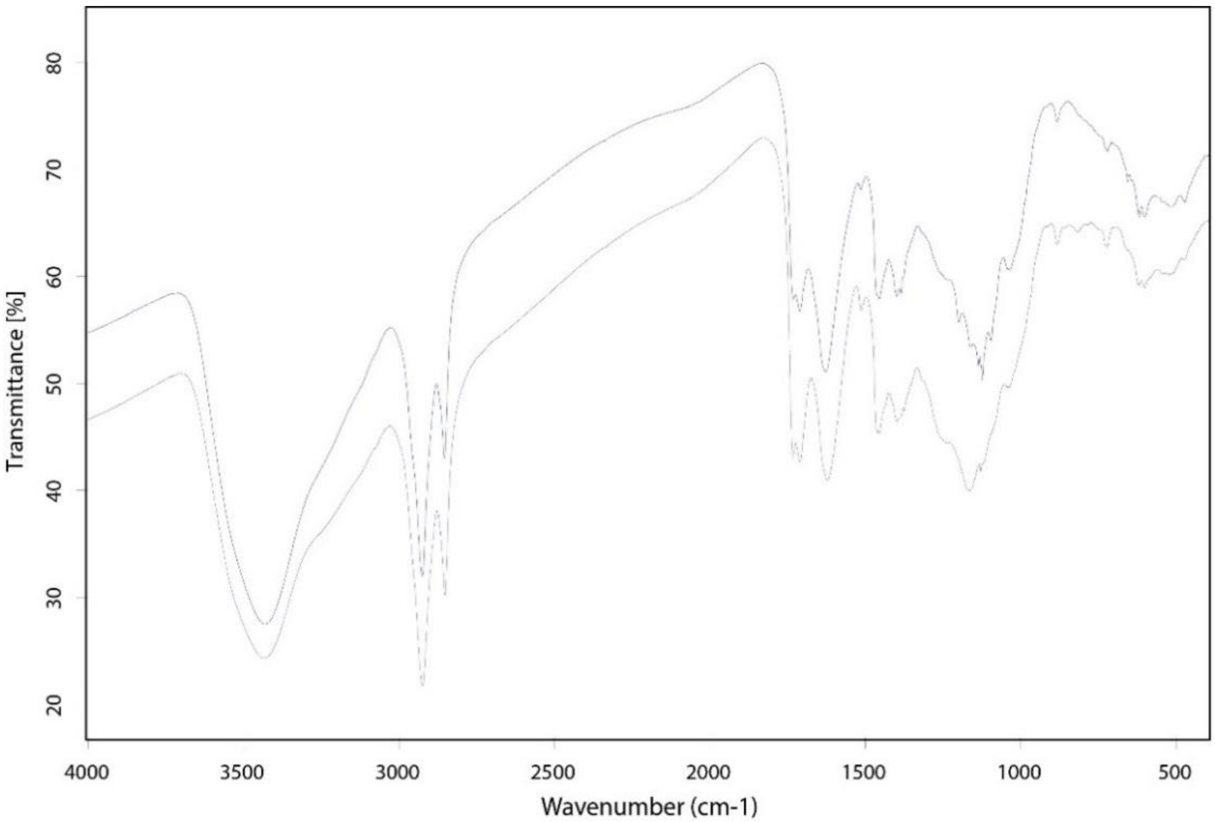

Figure S6. Complete transmission infrared spectra of the two Königsaue artefacts between 4000-400 cm^{-1} before spectral treatment. Upper spectrum: Königsaue 1, lower spectrum: Königsaue 2. Spectra are not offset so that the transmittance values are correct.

To increase to robustness of our analysis, we conducted a second PCA on a reduced set of 28 variables that were taken to be the peaks of the strongest positive and negative bands on the first derivative spectrum (Figure S7). While not separating the two aboveground techniques (condensation and cobble groove) as well as the PCA on the complete data set, this analysis yielded the same result in terms of the identification of the Königsaue artifacts' production technique.

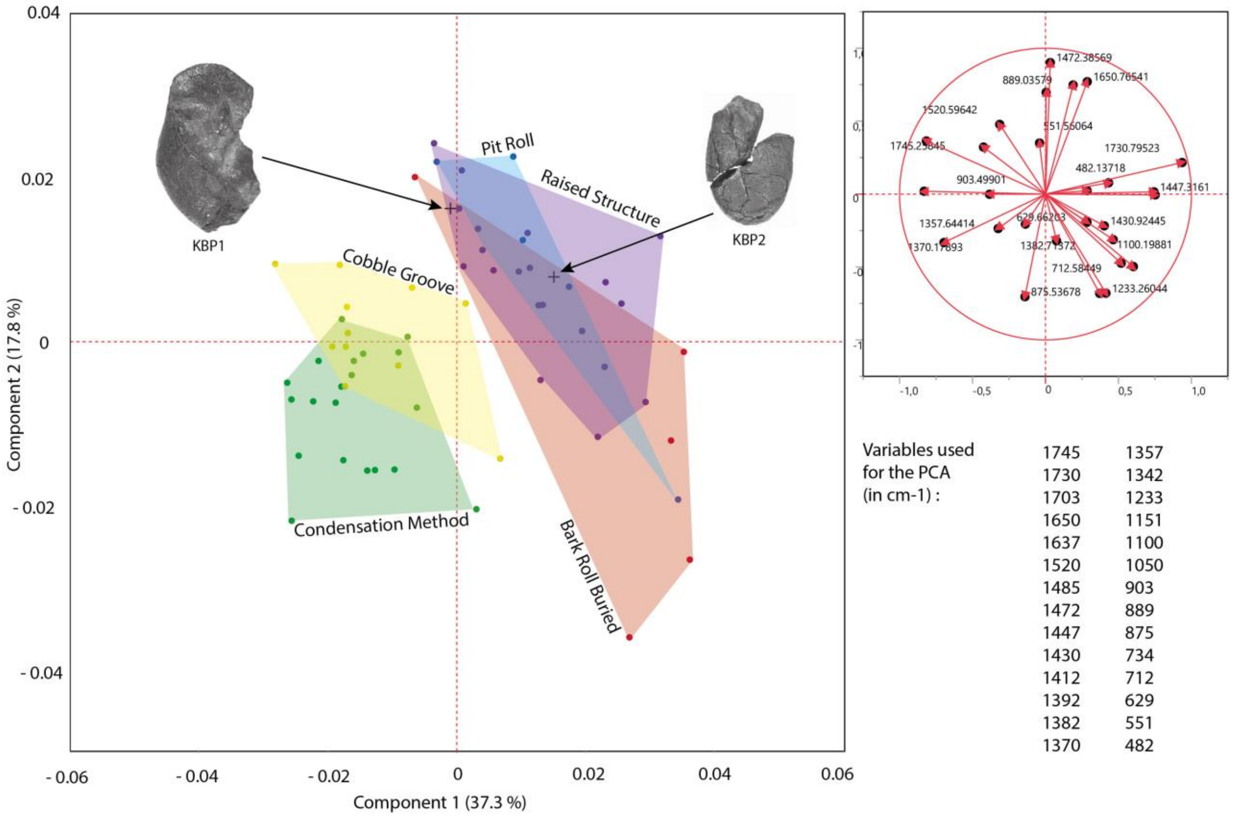

Figure S7. Principal Component Analysis (PCA) plot of first derivative data calculated from birch tar infrared spectra (between 1800 cm^{-1} and 400 cm^{-1}). Compared to the PCA plot in the main text, this plot was generated from a reduced set of 28 variables. Variables were chosen as maximum and minimum peaks in the first derivative spectra.

Supplementary information on our GC-MS analysis

Chemicals used for the analyses

Dichloromethane (Fisher scientific) and methanol (Carbo Erba) were HPLC grade and were used without further purification. Pyridine, N,O-bis(trimethylsilyl)trifluoroacetamide (BSTFA) and diatomaceous earth (Celite® 545) were purchased from Sigma Aldrich. Only dichloromethane cleaned glassware and above all no plastic material was used to avoid any contamination.

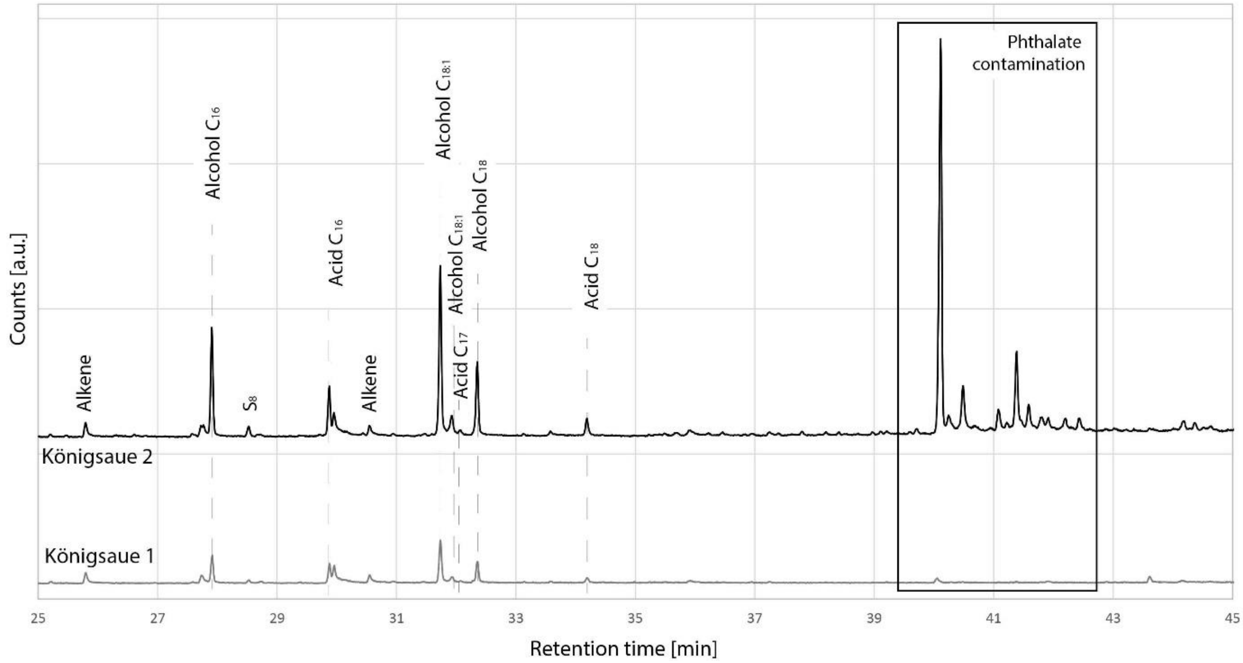

Figure S8. Chromatograms of the two Königsaue artefacts between 25-45 min. Note that only the younger Königsaue 2 is contaminated with phthalates (plasticizers).

Table S3. List of compounds identified by GC-MS in their trimethylsilylated form. Compounds are listed in ascending order of their retention time in the two Königsaue birch tar artefacts. Peak assignment was based on interpretation of mass spectra obtained with the OpenLab software and comparisons with spectra available in literature and NIST library 2.0.

Retention time (min)	Markers family	Compound	Königsaue 1	Königsaue 2
25.81	linear structure	alkene	√	√
27.92	linear structure	alcohol C_{16}	√	√
28.50	elemental sulphur	S_8	-	√
29.88	linear structure	acid C_{16}	√	√
29.96	linear structure	unsaturated alcohol	√	√
30.56	linear structure	alkene	√	√
31.73	linear structure	alcohol $C_{18:1}$	√	√
31.91	linear structure	alcohol $C_{18:1}$	√	√
32.06	linear structure	acid C_{17}	-	√
32.35	linear structure	alcohol C_{18}	√	√
34.19	linear structure	acid C_{18}	√	√
40.11	phtalate	phtalate	√	√
40.49	phtalate	phtalate	-	√
41.09	phtalate	phtalate	-	√
41.38	phtalate	phtalate	-	√
48.69	lupane derivative	lupa-2,20(29)-diene	√	√

50.33	linear structure	alcohol C_{28}	√	√
51.47	lupane derivative	lupa-2,20(29)-dien-28-ol	√	√
51.93	lupane derivative	allobetul-2-ene	√	√
53.11	plant sterol	β-sitosterol	√	√
53.27	linear structure	alcohol C_{30}	√	√
53.46	lupane derivative	lupenone	√	√
53.76	lupane derivative	lupeol	√	√
55.71	lupane derivative	betulone	√	√
56.21	lupane derivative	betulin	√	√
56.59	lupane derivative	3-oxoallobetulane	√	√
56.90	lupane derivative	allobetulin	√	√

√ : identified - : not present
Cx:y linear structure with x carbon atoms and y unsaturations

Supplementary images obtained by microCT scanning

CT-scans were recorded with a Phoenix v-tome-x s scanner (General Electric, Frankfurt am Main) and selecting a resolution of about 4.7 microns. The reconstructed volumetric data (.vol) was sliced and the ISO surface of the pieces generated, using the Avizo Lite software.

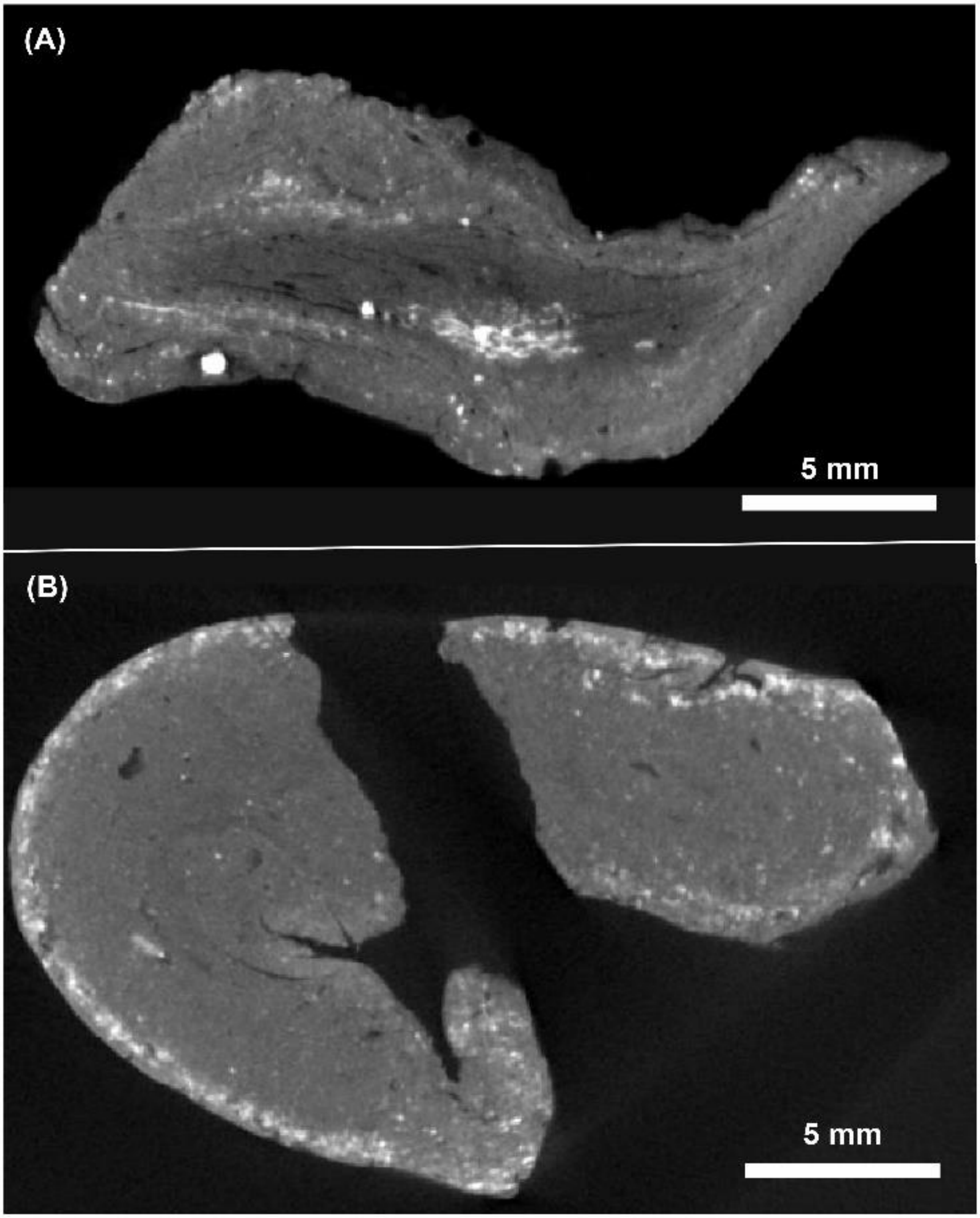

Fig. S9. MicroCT slice of Königsaue 1 (A) and 2 (B). The inclusions in Königsaue 1 appear to be small, rounded, and about 2.15 times denser than the surrounding tar. They are likely sand inclusions. Königsaue 2 shows a denser outer crust that is most likely due to taphonomy but no sand inclusions.

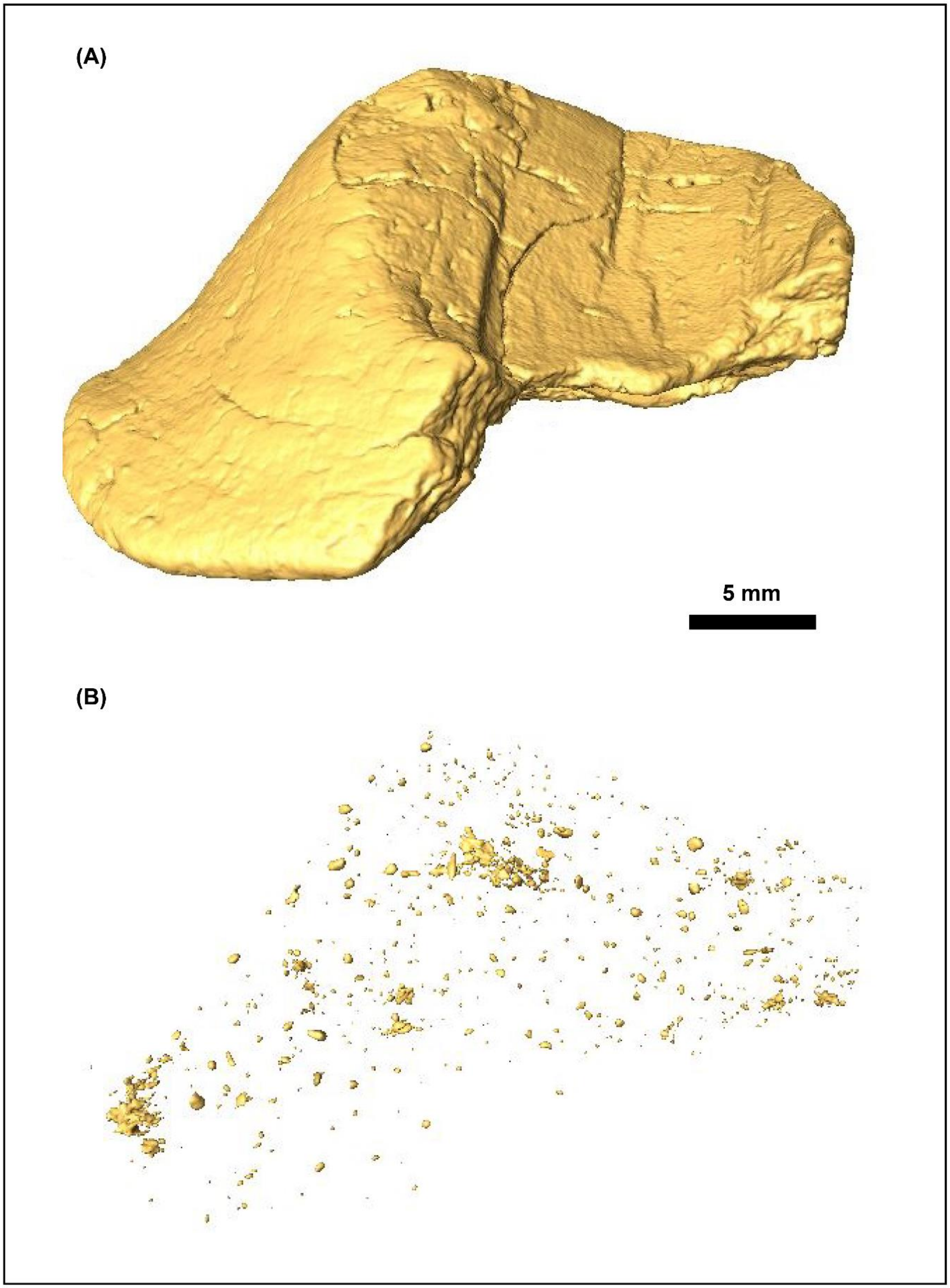

Figure S10. Extracted ISO surface of Königsaure 1 (A) and its segmented sediment inclusions (B).

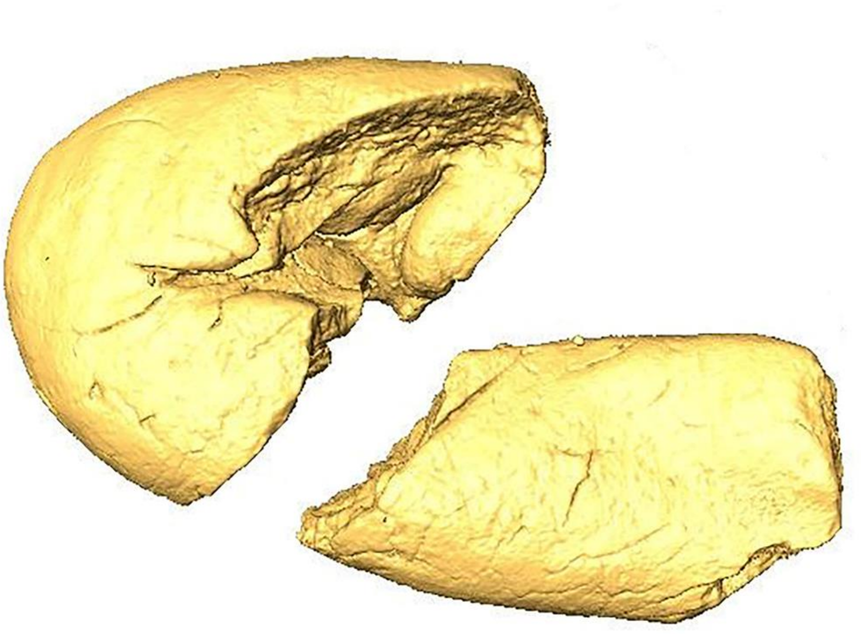

Figure S11. Extracted ISO surface of Königsaue 2.

Supplementary references

Berner, U., and H. Streif. 2000. *Klimafakten: der Rückblick-ein Schlüssel für die Zukunft* (Schweizerbart).

Boëda, Eric, Stéphanie Bonilauri, Jacques Connan, Dan Jarvie, Norbert Mercier, Mark Tobey, Hélène Valladas, Heba al Sakhel, and Sultan Muhesen. 2008. 'Middle Palaeolithic bitumen use at Umm el Tlel around 70 000 BP', *Antiquity*, 82: 853-61.

Degano, Ilaria, Sylvain Soriano, Paola Villa, Luca Pollarolo, Jeannette J. Lucejko, Zenobia Jacobs, Katerina Douka, Silvana Vitagliano, and Carlo Tozzi. 2019. 'Hafting of Middle Paleolithic tools in Latium (central Italy): New data from Fossellone and Sant'Agostino caves', *PLoS ONE*, 14: e0213473.

Grünberg, JM, H Gratsch, U Baumer, and J. Koller. 1999. 'Utersuchung der mittelpaläolithischen "Harzreste" von Königsaue, Ldkr. Aschersleben-Stassfurt', *Jahresschrift für mitteldeutsche Vorgeschichte*, 81: 7-38.

Hauck, Thomas C., Jacques Connan, Armelle Charrié-Duhaut, Jean-Marie Le Tensorer, and Heba al Sakhel. 2013. 'Molecular evidence of bitumen in the Mousterian lithic assemblage of Hummal (Central Syria)', *Journal of Archaeological Science*, 40: 3252-62.

Koch, Tabea J., and Patrick Schmidt. 2022. 'A new method for birch tar making with materials available in the Stone Age', *Scientific Reports*, 12: 413.

Koller, Johann, Ursula Baumer, and Dietrich Mania. 2001. 'High-tech in the middle Palaeolithic: Neandertal-manufactured pitch identified', *European Journal of Archaeology*, 4: 385-97.

Kozowyk, P. R. B., M. Soressi, D. Pomstra, and G. H. J. Langejans. 2017. 'Experimental methods for the Palaeolithic dry distillation of birch bark: implications for the origin and development of Neandertal adhesive technology', *Scientific Reports*, 7: 8033.

Kurzweil, Andreas, and Dieter Todtenhaupt. 1992. 'Technologie der Holzteergewinnung', *Acta Praehistorica et Archaeologica*, 23: 63-91.

Mania, D. 1999. '125 000 Jahre Klima-und Umweltentwicklung im mittleren Eibe-Saale-Gebiet', *Hercynia-Ökologie und Umwelt in Mitteleuropa*, 32: 1-97.

———. 2015. 'Messer aus den mittelpaläolithischen Uferstationen von Königsaue (Nordharzvorland) und Neumark-Nord (Geiseltal)/Knives from the Middle Palaeolithic statinos of Königsaue (Nordharzvorland) and Neumark Nord (Geiseltal).', *Anthropologie*, 53: 31-60.

Mania, D. . 2006. 'Stratigraphie, Klima-und Umweltentwicklung der letzten 400 000 Jahre im Saalegebiet und Harzvorland (Forschungsstand 2006)', *Hercynia-Ökologie und Umwelt in Mitteleuropa*, 39: 155-94

Mania, D., and V Toepfer. 1973. *Königsaue: Gliederung, Ökologie und mittelpaläolithische Funde der letzten Eiszeit* (EB Deutscher Verlag der Wissenschaften).

Mazza, Paul Peter Anthony, Fabio Martini, Benedetto Sala, Maurizio Magi, Maria Perla Colombini, Gianna Giachi, Francesco Landucci, Cristina Lemorini, Francesca Modugno, and Erika Ribechini. 2006. 'A new Palaeolithic discovery: tar-hafted stone tools in a European Mid-Pleistocene bone-bearing bed', *Journal of Archaeological Science*, 33: 1310-18.

Niekus, Marcel J. L. Th, Paul R. B. Kozowyk, Geeske H. J. Langejans, Dominique Ngan-Tillard, Henk van Keulen, Johannes van der Plicht, Kim M. Cohen, Willy van Wingerden, Bertil van Os, Bjørn I. Smit, Luc W. S. W. Amkreutz, Lykke Johansen, Annemieke Verbaas, and Gerrit L. Dusseldorp. 2019. 'Middle Paleolithic complex technology and a Neandertal tar-backed tool from the Dutch North Sea', *Proceedings of the National Academy of Sciences*: 201907828.

Pawlik, A., and J. Thissen. 2011. 'Hafted armatures and multi-component tool design at the Micoquian site of Inden-Altdorf, Germany.', *Journal of Archaeological Science*, 38: 1699-708.

Pawlik, Alfred F. 2004. 'Lithics in Action.' in E. A. Walker, F. Wenban-Smith and F. Healey (eds.), *Papers from the Conference on Lithic Studies in the Year 2000* (Oxbow Books: Oxford).

Picin, A. 2016. 'Short-term occupations at the lakeshore: a technological reassessment of the open–air site Königsaue (Germany)', *Quartär–Internationales Jahrbuch zur Erforschung des Eiszeitalters und der Steinzeit*, 63: 7-32.

Rots, V. 2015. Nicholas Conard and Anne Delagnes (eds.), *Settlement Dynamics of the Middle Paleolithic and Middle Stone Age, Volume IV* (Kerns Verlag: Tübingen).

Ruebens, K. . 2013. 'Regional behaviour among late Neanderthal groups in Western Europe: a comparative assessment of late Middle Palaeolithic bifacial tool variability', *Journal of Human Evolution*, 65: 341-62.

Schmidt, Patrick, Matthias Blessing, Maxime Rageot, Radu Iovita, Johannes Pfleging, Klaus G. Nickel, Ludovic Righetti, and Claudio Tennie. 2019. 'Birch tar production does not prove Neanderthal behavioral complexity', *Proceedings of the National Academy of Sciences*, 116: 17707.

Sykes, RM Wragg. 2015. 'To see a world in a hafted tool: birch pitch composite technology, cognition and memory in Neanderthals.' in, *Settlement, Society and Cognition in Human Evolution* (Cambridge University Press).
Wiśniewski, A. . 2014. 'The beginnings and diversity of Levallois methods in the early Middle Palaeolithic of Central Europe. ', *Quaternary International* 326: 364-80.
